# H3K18 lactylation potentiates microglial polarization via the TLR4 pathway in diabetes-induced cognitive impairment

**DOI:** 10.1172/jci.insight.188077

**Published:** 2025-11-04

**Authors:** Ying Yang, Fei Chen, Lulu Song, Liping Yu, Jinping Zhang, Bo Zhang

**Affiliations:** 1 Department of Endocrinology, China-Japan Friendship Hospital, Beijing, China.; 2 Department of Endocrinology, Peking University First Hospital, Beijing, China.

**Keywords:** Endocrinology, Metabolism, Neuroscience, Diabetes

## Abstract

The present study aimed to explore the role and possible underlying mechanisms of histone lactylation (Kla) modifications in diabetes-associated cognitive impairment (DACD). In this study, behavioral tests, H&E staining, and immunohistochemistry were used to evaluate cognitive function and the extent of cerebral tissue injury. We quantified the levels of lactic acid and pan-lysine Kla (Pan-Kla) in the brains of type 2 diabetes mellitus (T2DM) mice and in high glucose–treated microglia. We also identified all Kla sites in isolated microglia. Gene Ontology (GO) enrichment analysis and Kyoto Encyclopedia of Genes and Genomes (KEGG) pathway analysis were subsequently conducted to identify the functions and pathways that were enriched at the differentially expressed modification sites. Cleavage under targets and tagmentation (CUT&Tag) technology was used to identify candidate genes that are regulated by histone H3 lactylated at Lys-18 (H3K18la). siRNA and H3K18R mutant sequences were used to knock down crucial components in key signaling pathways to assess the effects of histone Kla on microglial polarization. We found that lactic acid levels were significantly greater in the brains of T2DM mice and high glucose–treated microglia than in those of their corresponding controls, which increased the level of Pan-Kla. We discovered that lactate can directly stimulate an increase in H3K18la. The global landscape of the lactylome reveals information about modification sites, indicating a correlation between the upregulation of H3K18la and protein Kla and Toll-like receptor (TLR) signaling. CUT&Tag demonstrated that enhanced H3K18la directly stimulates the NF-κB signaling pathway by increasing binding to the promoter of TLR4, thereby promoting M1 microglial polarization. The present study demonstrated that enhanced H3K18la directly stimulates TLR4 signaling to promote M1 microglial polarization, thereby facilitating DACD phenotypes. Targeting such loop may be a potential therapeutic approach for the treatment of DACD.

## Abstract 2

H3K18la/TLR4 axis orchestrates neuroinflammation by regulating microglial polarization, thereby fostering the emergence of diabetes-associated cognitive decline phenotypes.

## Introduction

Diabetes and cognitive dysfunction are chronic disorders with a high global prevalence. Diabetic patients face a markedly elevated susceptibility to cognitive deficits, characterized by diminished memory, reduced cognitive flexibility, and, in severe cases, intellectual impairment and behavioral abnormalities. This leads to a reduction in patients’ self-care capacity and greater reliance on caregivers, thereby accelerating the progression of diabetes and establishing a detrimental feedback loop (1). The incidence of diabetes-associated cognitive decline (DACD) is as high as 13.5%, and it exceeds 24.2% in those aged 75 years and above (2). Consequently, this condition intensifies the financial strain on both families and society, emerging as a critical public health challenge that demands prompt and effective intervention.

The pathogenesis of DACD is multifactorial. Under hyperglycemic conditions, neuronal glucose metabolic pathways are altered, leading to impairments in mitochondrial function and aberrant cellular energy production, which subsequently elevates lactate generation (3). Lactate can provide energy for neuronal cells, protect damaged axons, and promote axonal regeneration. Sustained and excessive lactate buildup disrupts the intracellular acid-base equilibrium, exacerbates oxidative damage, induces neuronal apoptosis, and adversely affects normal neuronal function through multiple pathways (4–5). As a precursor to protein lactylation (Kla) (6), lactate facilitates Kla modifications and also contributes to the regulation of glycolytic processes (7), macrophage polarization (8), tumor proliferation (9), and other important cellular activities. It is widely present in various cells and causes various diseases in the body (10). Lactate also participates in the regulation of the nervous system through Kla in neuronal cells. According to a recent investigation, externally supplied lactate induces region-specific protein Kla within the brain; this effect occurs widely in neuronal cells in the brain, including microglia (11). Studies focusing on astrocytic protein Kla have revealed elevated Kla levels near amyloid-β (Aβ) plaques in Alzheimer disease (AD) mouse models. Furthermore, Kla is associated with the proinflammatory activation of astrocytes (12). Kla modifies not only histone proteins but also numerous nonhistone proteins. Histone Kla involves the addition of lactyl moieties to lysine residues on histone molecules, altering the charge properties and structure of histones. This modification affects their interaction with DNA, thereby regulating transcriptional activity and contributing prominently to the maintenance of cellular metabolic and functional homeostasis (13). Nevertheless, the function of histone Kla in the context of DACD remains poorly documented.

Experimental work undertaken here involved the construction of a DACD mouse model, through which we found an association linking aberrant lactic acid accumulation to modifications in histones via Kla in microglia within the hippocampus, ultimately resulting in impaired cognitive function. Through our investigation, specific Kla sites on histones were identified within the hippocampus, notably observing a marked increase in histone H3 lactylated at Lys-23 (H3K23la) in DACD mouse microglia. By leveraging the cleavage under targets and tagmentation (CUT&Tag) assay, we further determined enrichment of H3K18la at promoter regions of genes linked to the Toll-like receptor 4 (TLR4) signaling cascade. Notably, when high glucose–treated (HG-treated) microglia were treated with lactate dehydrogenase (LDH) inhibitors, there was a marked reduction in lactate concentrations, which in turn led to reduced H3K18la modification and suppression of the TLR4 pathway. Our findings underscore the direct stimulatory effect of enhanced H3K18la on the TLR4 signaling pathway, which exacerbates neuroinflammation and ultimately contributes to the manifestation of DACD phenotypes.

## Results

### Hyperglycemia can cause cognitive impairment and brain tissue damage.

**Figure 1:**
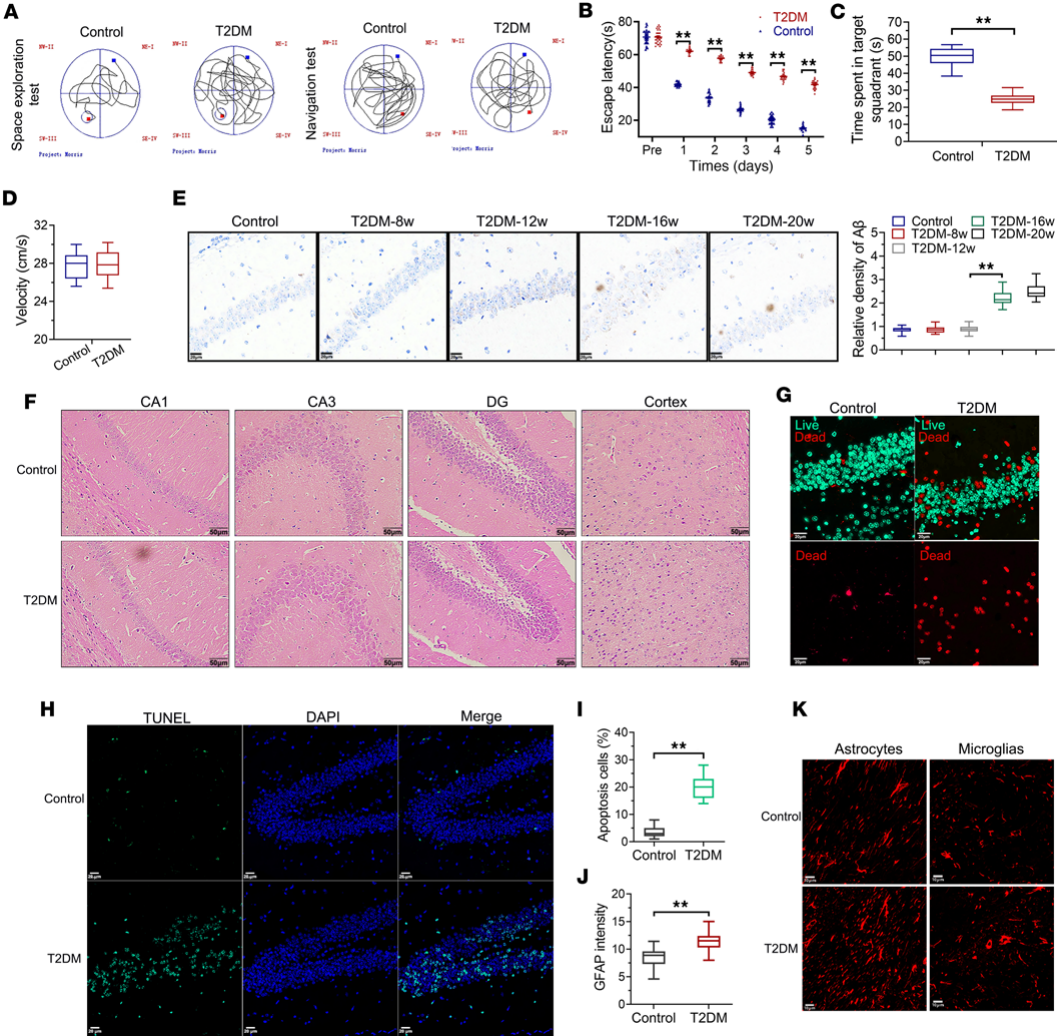
Hyperglycemia can cause cognitive impairment and brain tissue damage. (**A**) Representative tracks of the distances moved in the Morris water maze (MWM) in the different groups. (**B**) Analysis of the escape latency data from the positioning navigation tests. (**C**) Time spent in target quadrant in space exploration tests. (**D**) Swimming speeds of mice in the MWM task. (**E**) Aβ immunohistochemistry in hippocampal tissues and quantification. Scale bars: 20 μm. (**F**) Representative images of H&E staining in various brain regions (original magnification, ×400; scale bars: 50 μm). (**G**) Immunostaining representative of tissue viability at the sectioning stage, depicting live (green) and dead (red) zones. Scale bars: 20 μm (all panels). (**H** and **I**) TUNEL assay and statistical results of TUNEL^+^ cells. (**J** and **K**) Fluorescence images of hippocampal sections expressing GFAP in microglial cells or astrocytes. Scale bars: 10 μm. *n* = 3 per group. Data are expressed as mean ± SD. Statistical analysis was performed using 2-tailed Student’s *t* tests (**B**–**D**, **I**, and **J**) or 2-way ANOVA with Tukey’s test for differences among groups (**E**). **P < 0.01. T2DM, type 2 diabetes mellitus.

We performed Morris water maze tests, which are used to assess spatial learning and memory. In the Morris water maze task involving place and cue navigation, compared with age-matched controls, mice with type 2 diabetes mellitus (T2DM) exhibited prolonged latency to locate the platform (Figure 1, A and B). In subsequent probe trials, T2DM mice displayed a reduced inclination toward the trained target zone in comparison with the control group (Figure 1 A and C). Notably, both groups of mice exhibited similar swimming speeds (Figure 1D). Cerebral deposition of Aβ plaques is a remarkable contributor to cognitive impairment. As shown in Figure 1E, Aβ deposition became detectable in T2DM models starting at 16 weeks after induction, with no visible accumulation observed at earlier time points (8 or 12 weeks). Our study utilized hematoxylin and eosin (H&E) staining to assess neuronal morphology and quantity. In control mice, hippocampal cells displayed organized arrangements without morphological anomalies. Conversely, hyperglycemia induced neuronal damage and loss in the hippocampus and cortex of T2DM mice compared with controls (Figure 1F). Microscopic analysis revealed irregular cellular patterns and pyknotic nuclei in T2DM mice, which were notably pronounced in the hippocampus. In addition to these, cells subjected to T2DM-relevant metabolic stress demonstrated significantly elevated mortality rates relative to normoglycemic controls (Figure 1G). TUNEL assays showed that T2DM significantly increased TUNEL-positive cells compared with the control mice (Figure 1, H and I). In tests analyzing astro- and microgliosis, immunostaining demonstrated substantially elevated glial fibrillary acidic protein (GFAP) fluorescence intensity in T2DM astrocytes and microglial cells compared with the control group (Figure 1, J and K). Hippocampal impairment is an important pathological basis for cognitive impairment in individuals with T2DM. The above findings establish that T2DM can cause neuronal injury in the hippocampus across multiple pathological dimensions.

### Hyperglycemia elevated lactate levels and Pan-Kla levels in the cortex and hippocampus.

**Figure 2:**
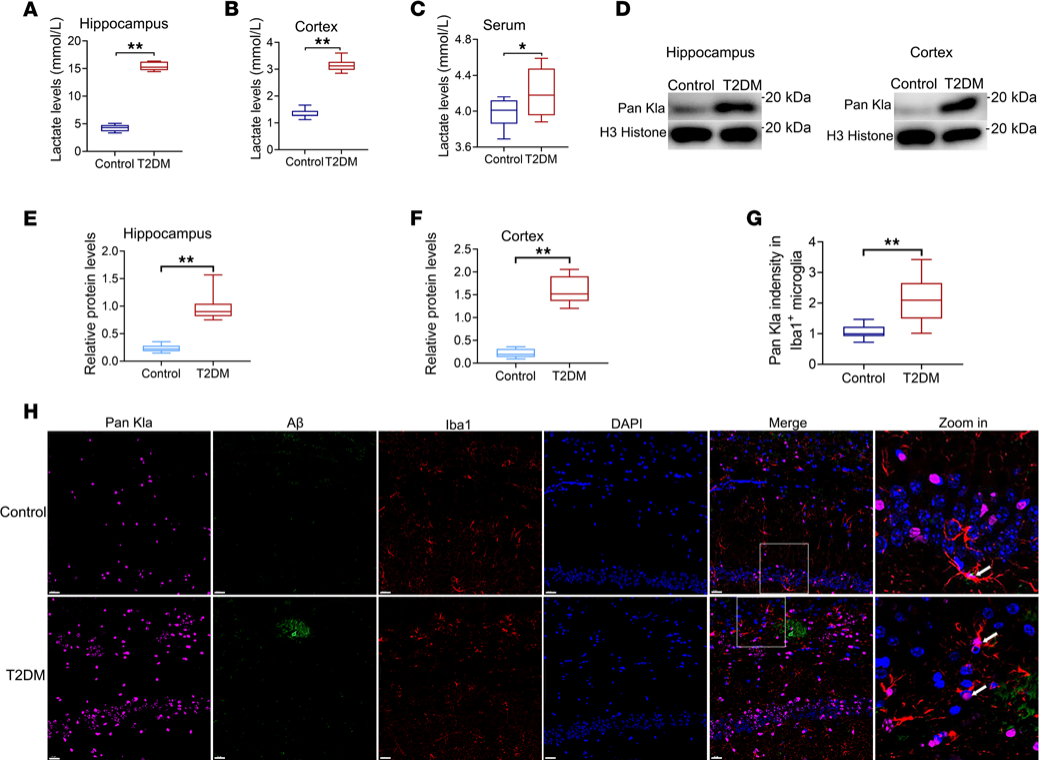
Lactate levels and Pan-Kla are increased in the cortex and the hippocampus of T2DM mice. Lactate levels in hippocampus (**A**), cortex (**B**), and serum (**C**) of mice in different groups. (**D**) Western blotting analysis of Pan-Kla in the cortex and hippocampus of mice; graphs are representative of 3 independent experiments. Quantification of Western blot images in hippocampus (**E**) and cortex (**F**). (**G**) Quantification of Pan-Kla intensity. (**H**) Representative images of Pan-Kla costained for Aβ^+^ plaques and microglia (Iba1^+^) in the hippocampal CA1 region of mice (scale bars: 10 μm). *n* = 3 per group. Data are expressed as mean ± SD. Statistical analysis was performed using 2-tailed Student’s *t* tests (**A**–**C** and **E**–**G**). *P < 0.05, **P < 0.01. Kla, lactylation.

To evaluate the metabolic reprogramming of the hippocampus and cortical tissue from oxidative phosphorylation (OXPHOS) to glycolysis, we measured lactate levels in streptozotocin- (STZ-) and high-fat diet–induced (HFD-induced) diabetic mice (a well-established T2DM model). Quantification of lactate demonstrated that lactate levels were significantly elevated in both the hippocampal/cortical tissues and serum of T2DM mice (Figure 2, A–C). Moreover, Western blot analysis revealed that pan-lysine Kla (Pan-Kla) levels were also dramatically elevated in both the hippocampal and cortical tissues of T2DM mice (Figure 2, D–F). Microglia play a defensive role in the frontline response to injury. Therefore, we focused on microglia in a follow-up study. To examine changes in proteins with Kla modifications in microglia, we performed immunofluorescent costaining of Pan-Kla along with antibodies against a marker for microglia (Iba1). Immunofluorescence analysis revealed Pan-Kla/Iba1 colocalization, and more cells displayed Pan-Kla fluorescence in the hippocampus of T2DM mice (Figure 2, G and H). The Pan-Kla signal intensity was significantly greater in the Aβ plaque–adjacent microglia of T2DM mice than in those of control mice.

### Global landscape of the lactylome in isolated microglial cells.

**Figure 3:**
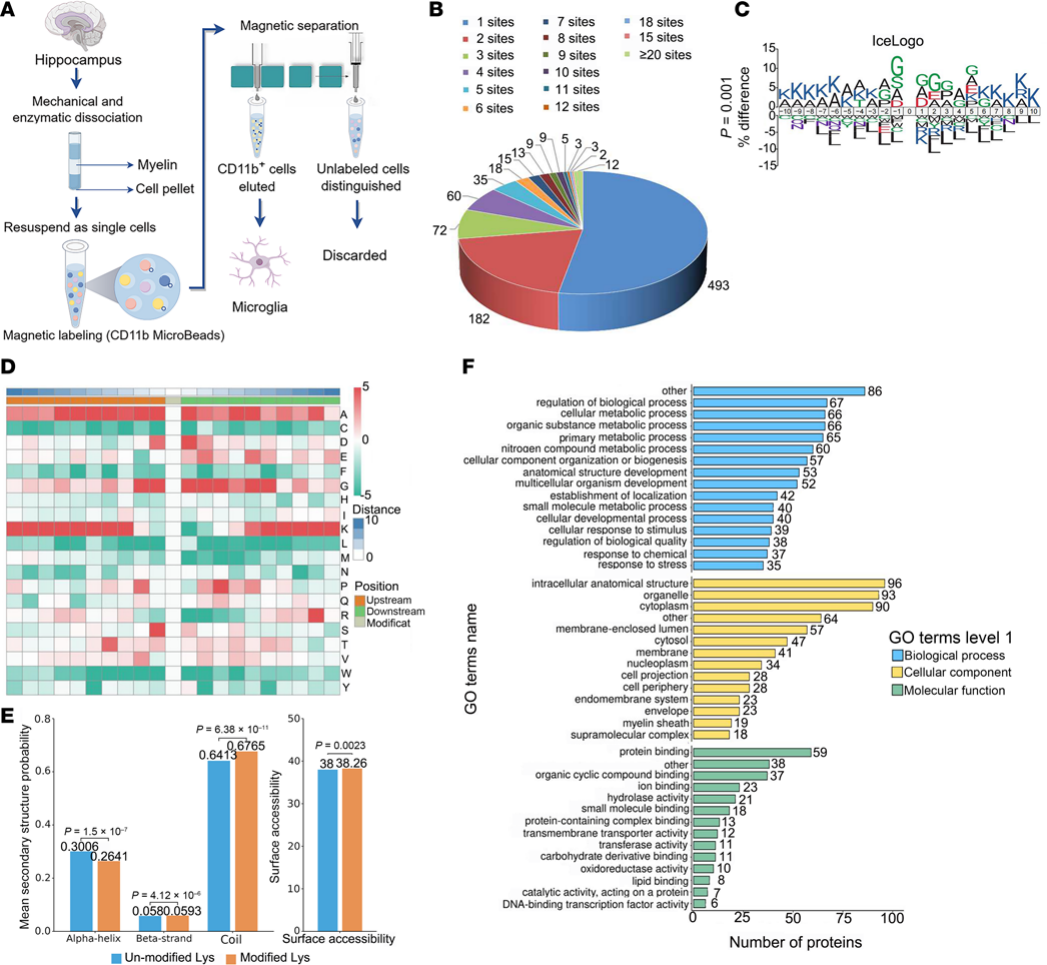
Identification of Kla proteome profile in isolated microglia. (**A**) Schematic of microglia isolated from adult brains of T2DM and control mice. (**B**) Pie chart illustrating the distribution of the quantity of identified Kla sites per protein. (**C**) Motif analysis for the identified lactylated proteins. (**D**) The iceLogo representation displays the flanking sequence preferences for all Kla sites located between the upstream +10 position and the downstream –10 position. (**E**) Distribution of all lysines and Kla lysines in the structured regions of proteins. (**F**) Bar graphs illustrating the ontology annotations representative of the Kla proteome enrichment. Statistical analysis was performed using 2-tailed Student’s *t* tests (**C** and **E**).

To comprehensively understand the lactylome, which encompasses both histone and nonhistone Kla, we conducted a proteomic analysis specifically targeting Kla. In brief, microglial cells obtained from both T2DM and control mice were isolated using magnetic separation techniques (Figure 3A). Sorted cells were confirmed by immunofluorescence and revealed very few cellular protrusions (Supplemental Figure 1, A and B; supplemental material available online with this article; https://doi.org/10.1172/jci.insight.188077DS1). A minimum of 1 × 10^7^ cells were collected. Purity check was performed by flow cytometry and showed purity to be 98% or greater (Supplemental Figure 1, C and D). Cells maintained essential stability during the sorting procedure (Supplemental Figure 1, E and F). A total of 2424 Kla sites were identified across 931 proteins, with 2254 sites from 871 proteins quantified. Among these proteins, 496 (52.95%) had a single Kla site, 182 (19.55%) had 2, and 1804 (27.5%) had more than 3 (Figure 3B). We next analyzed the amino acids flanking the identified Kla sites using iceLogo (Figure 3C). Amino acid analysis of the flanking Kla sites identified using iceLogo revealed enrichment of serine and alanine at positions –1 and +1, respectively (Figure 3D). Structural analysis via NetSurfP software (https://services.healthtech.dtu.dk/service.php?NetSurfP-2.0) indicated that approximately 26.41% of Kla sites were in α-helices, 5.93% were in β-strands, and the remaining 67.65% were in irregular coils. There was no apparent difference compared to total protein lysine residues, suggesting that there was no structural preference for Kla, at least in isolated microglial cells. However, the average surface accessibility of the Kla protein was significantly less than that of the total protein lysine residues, indicating preferential localization within protein structures (Figure 3E). Gene Ontology (GO) analysis revealed that lactylated proteins participate in various cellular processes, including cellular regulation, metabolism, and response to stimuli (Figure 3F).

### Quantitative analysis of the Kla proteome in isolated microglial cells from different groups.

**Figure 4:**
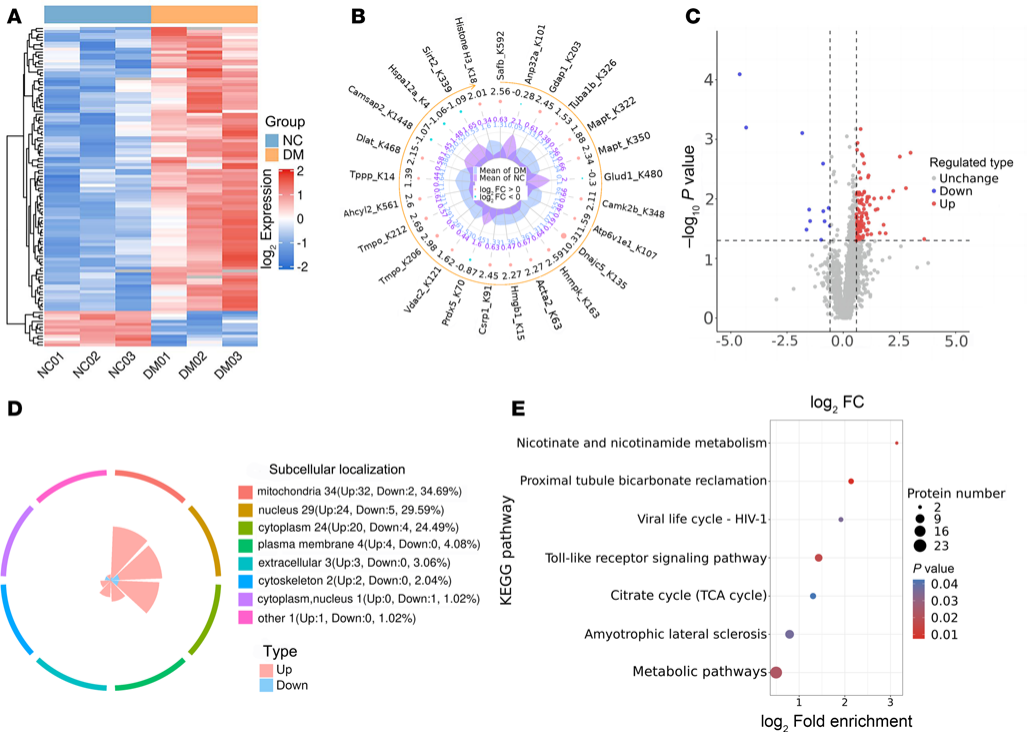
Quantification of the lactylome in microglia in response to different glycemic status. (**A**) Heatmap showing the changes in the quantifiable Kla sites between negative control and T2DM groups. (**B**) Radar maps of the top upregulated and downregulated Kla sites. (**C**) Scatter plot showing changes in the quantifiable Kla sites between negative control and T2DM groups. (**D**) The subcellular localization of the differentially modified proteins. (**E**) Bubble diagram showing KEGG pathway enriched with upregulated and downregulated lactylated proteins.

The threshold for significant Kla changes between the T2DM and control groups was a ratio above 1.5 or below 0.67. Analysis revealed the upregulation of 96 Kla sites in 86 proteins and the downregulation of 12 Kla sites in 12 proteins in the microglia of T2DM mice (Figure 4, A–C). These proteins have been observed to localize to diverse subcellular compartments, such as the mitochondria, cytoplasm, and nucleus (Figure 4D). The chord diagram depicts the 4 most enriched Kyoto Encyclopedia of Genes and Genomes (KEGG) pathways and the connections between Kla sites and these pathways. KEGG enrichment analysis revealed that the significantly differentially expressed lactylated proteins were primarily involved in pathways such as TLR signaling, citrate cycle (TCA cycle), nicotinate and nicotinamide metabolism, amyotrophic lateral sclerosis, proximal tubule bicarbonate reclamation, HIV-1 viral life cycle, and metabolic pathways (Figure 4E).

### Increased levels of H3K18la are observable in microglial cells within the hippocampal tissues of T2DM mice and in microglia treated with HG.

**Figure 5:**
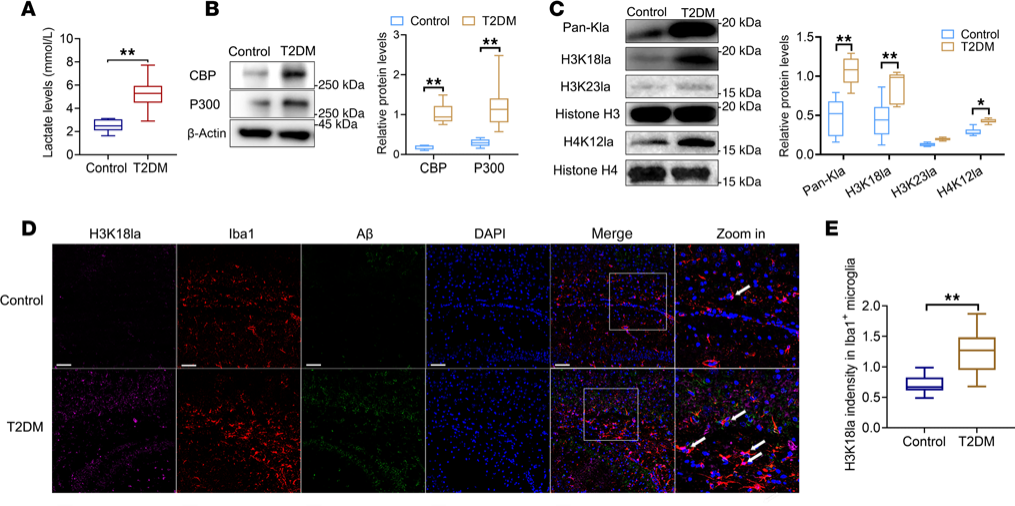
Elevated levels of H3K18 Kla can be observed in microglia within the hippocampus of T2DM mice. (**A**) Lactate levels in microglia within the hippocampus in mice. *n* = 3. (**B**) Western blotting analysis of CBP and P300 in microglia in the hippocampus of mice. (**C**) Western blotting analysis of Pan-Kla, H3K18la, H4K12la, and H3K23la in microglia in the hippocampus of mice. *n* = 3. (**D**) Representative images of H3K18la costained for Aβ plaques and Iba1 in the hippocampal CA1 region of mice (scale bars: 20 μm). More than 5 fields of view were randomly selected to be analyzed. (**E**) Quantification of H3K18la intensity in Iba^+^ microglia. *n* = 3 per group. Data are expressed as mean ± SD. Statistical analysis was performed using 2-tailed Student’s *t* tests. *P < 0.05, **P < 0.01. NC, negative control; HG, high glucose; Aβ, amyloid-β.

The proteomic results were further validated by immunoblot analyses and immunofluorescent staining. The level of lactate in microglial cells within the hippocampus was substantially greater in T2DM mice than in control mice (Figure 5A). CREB-binding protein (CBP) and P300 are histone acetyltransferases that also play roles in protein Kla (14). Western blot analysis revealed enhanced expression levels of CBP and P300 in microglia in the hippocampus of T2DM mice compared with those in control mice (Figure 5B). The results also showed that H3K18la was elevated in microglia in hippocampal tissues from T2DM mice (Figure 5C). Furthermore, we conducted immunofluorescent staining for H3K18la, along with markers for microglia (Iba1) and Aβ plaques, in postmortem brain sections from mice. We found that H3K18la was notably greater in microglia adjacent to plaques in the T2DM group than in microglia in the control group (Figure 5, D and E).

For in vitro experiments, 50 mM oxamate was added to suppress LDH activity, and siP300 was used to suppress P300 expression (Supplemental Figure 2, A and B). We conducted experiments in cultured primary microglial cells and HMC3 cells, employing 3 distinct glucose concentration media (HG, moderate, and low glucose) to culture primary microglia and HMC3 cells. As demonstrated in Supplemental Figure 3, A and B, both moderate-glucose and HG conditions resulted in significant reductions in cell viability compared with low-glucose controls, with the most pronounced decrease observed in the HG treatment group. Analysis of lactate metabolism revealed that intracellular lactate levels in moderate-glucose cultures remained comparable to those in low-glucose conditions. In contrast, HG exposure induced a substantial increase in lactate accumulation relative to the low-glucose group (Supplemental Figure 3C). Neither moderate- nor low-glucose culture conditions elicited detectable changes in Pan-Kla expression levels. In contrast, sustained exposure to HG triggered a marked upregulation of Pan-Kla expression compared with moderate-glucose controls (Supplemental Figure 3D). P300 siRNA did not cause a significant loss in cell viability up to 48 hours after exposure (Supplemental Figure 3, E and F). Higher lactate levels were detected in HG-treated primary microglia and HMC3 cells than in negative control cells. Relative lactate expression did not markedly decrease following P300 inhibition in either cell line, indicating that P300 did not affect lactate production. As shown in Supplemental Figure 4A, LDH suppression significantly inhibited lactate levels in primary microglia and HMC3 cells even after culture in HG medium. Histone Kla increased following incubation of primary microglia and HMC3 cells with sodium lactate (NaLa) (Supplemental Figure 5, A and C). Given the role of lactate as a precursor capable of inducing histone Kla, we hypothesized that there might be changes in histone Kla within the framework of cognitive decline associated with T2DM. We cultured cells with NaLa to promote Kla. Compared with control microglia, HG-treated microglia expressed higher levels of CBP and P300. In addition, NaLa-treated microglial cell lines exhibited considerably increased expression of CBP and P300 (Supplemental Figure 4, B and C). We also found that HG led to increased H3K18la expression in primary microglia, which was consistent with the results of the above animal experiments. Interestingly, there was also an increase in histone H3, which is lactylated at Lys-23 (H3K23la), and in histone H4, which is lactylated at Lys-12 (H4K12la), after HG treatment. As shown in Supplemental Figure 4, D and E, the presence of siP300 or oxamate in HG-treated primary microglia dramatically inhibited H3K18la and H4K12la but not H3K23la. In the HMC3 cell line, we found results similar to those we found in the primary microglial cells. This finding additionally confirms the crucial role of lactate in histone Kla. Immunofluorescence assays showed that HG increased the colocalization ratio of H3K18la to total Iba1 in primary microglia (Supplemental Figure 4F). Similarly, NaLa-treated primary microglia also exhibited a high ratio of H3K18la to total Iba1. Similar findings were also observed in HMC3 cells (Supplemental Figure 4, G and H).

### Genome-wide analysis of the transcriptional consequences of H3K18la in primary microglial cells.

**Figure 6:**
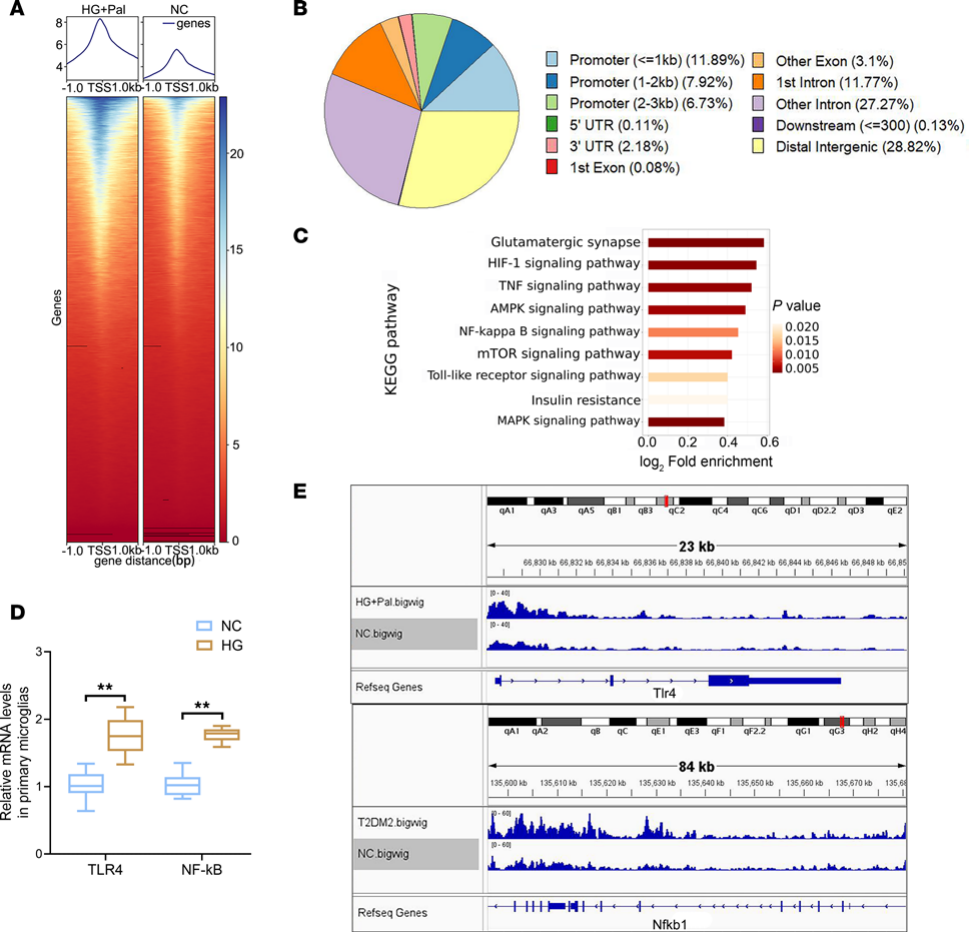
Analysis of the transcriptional consequences of H3K18la in HG-treated primary microglia. (**A**) Heatmap presenting the binding density of H3K18la with different H3K18la binding peaks in HG-treated primary microglial cells and control cells, ordered by signal strength. (**B**) Genome-wide distribution of upregulated H3K18la-binding peaks in HG-treated primary microglia. (**C**) KEGG analysis of enhanced H3K18la binding peaks at candidate target genes. (**D**) Genome browser tracks of H3K18la binding peaks at the representative target gene loci. (**E**) qPCR assays monitoring expression of the indicated genes in HG-treated primary microglial cells and control cells. *n* = 3 per group. Data are expressed as mean ± SD. Statistical analysis was performed using 2-tailed Student’s *t* tests. **P < 0.01. KEGG, Kyoto Encyclopedia of Genes and Genomes; TLR4, Toll-like receptor 4; NF-κB, nuclear factor κB.

Histone modifications can influence both the activation and inhibition of target genes through transcriptional regulation (15). To investigate the potential functional significance of H3K18la in HG-treated primary microglia, a genome-wide CUT&Tag analysis was performed to identify candidate genes that are regulated by H3K18la in microglia. CUT&Tag conducted with antibodies targeting H3K18la, followed by analysis using deepTools (https://github.com/deeptools/deepTools) and the ChIPseeker R package (https://github.com/ENCODE-DCC/chip-seq-pipeline2), demonstrated a significant increase in H3K18la peaks in primary microglia in the HG group compared with those in the control group (Figure 6A). In the comparison of microglia, 13,574 differential H3K18la binding peaks were identified, 11.89% of which were located within promoter sequences (≤1 kb), and with 7.92% of the peaks located within promoter sequences (1–2 kb) (Figure 6B). To explore the epigenetic effects of H3K18la in primary microglia treated with HG, we classified target genes with distinct H3K18la binding peaks at promoters into various KEGG pathways using GO analysis (Figure 6C). KEGG pathways, including the TLR, NF-κB, and mTOR signaling pathways, are implicated in insulin resistance and inflammation. Notably, the identified peaks highlighted genomic regions at glycolytic genes such as TLR4 and NF-κB, indicating elevated H3K18la levels at their promoters (Figure 6D). Real-time quantitative polymerase chain reaction (qPCR) analysis demonstrated a significant increase in the expression levels of these genes in primary microglia treated with HG compared with those in control cells (Figure 6E).

Transcriptome analysis was performed to delineate transcriptomic changes in sorted microglial cells. Transcriptomic profiling revealed distinct sample clustering in 2-dimensional PCA space, reflecting intrinsic gene expression covariation patterns, with tight aggregation of biologically analogous replicates (Supplemental Figure 6A). Expression distribution homogeneity was validated through integrated box-and-whisker plot–violin plot hybrid visualizations (Supplemental Figure 6, B and C). Comparative analysis identified 2010 significantly upregulated and 1984 downregulated differentially expressed genes (DEGs) in HG-treated microglia versus controls (Supplemental Figure 6, D–F). GO enrichment analysis of DEGs from the control versus T2DM group comparison revealed significant enrichment in multiple processes and functions. Key enriched terms included negative regulation of glial cell differentiation, regulation of inflammatory response, regulation of TLR4 signaling pathway, regulation of insulin secretion, DNA-binding transcription activator activity, modified amino acid binding, peptide binding, and nuclear receptor activity (Supplemental Figure 6G). Subsequent KEGG pathway analysis of the T2DM versus negative control (NC) comparison identified significant enrichment in the TLR signaling pathway, protein digestion and absorption, insulin secretion, T2DM, and others (Supplemental Figure 6H). Collectively, these findings suggest that H3K18la modification triggers the transcription of numerous genes encoding marker proteins involved in TLR4 signaling in primary microglial cells treated with HG.

### Inhibition of Kla reduced cognitive impairment and suppressed the TLR4 signaling pathway.

**Figure 7:**
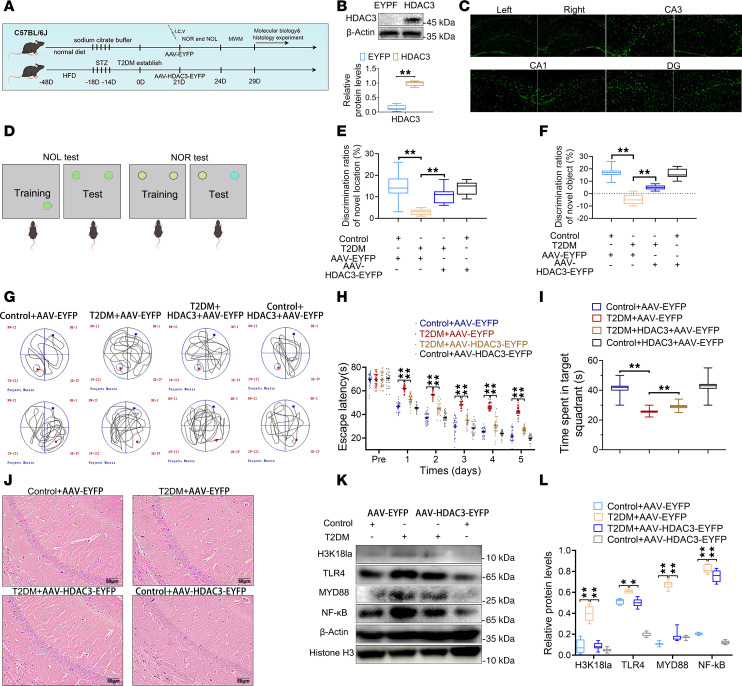
Inhibition of Kla of histone and H3K18 leads to TLR4/NF-κB suppression in vivo. (**A**) Workflow of animal experiments. (**B**) Western blotting analysis of HDAC3. (**C**) Validation of HDAC3 overexpression in the hippocampus. (**D**) Schematics of NOL and NOR tests. Results of NOL test (**E**) and NOR tests (**F**) (9 mice per group). (**G**) Morris water maze assay. (**H**) Analysis of the escape latency data from the positioning navigation tests. (**I**) Time spent in target quadrant in space exploration tests. (**J**) The results of H&E staining. *n* = 3. Scale bars: 50 μm. The bands (**K**) and quantification of the blots of TLR4/NF-κB signaling in microglial cells in the hippocampus (**L**); graphs are representative of 3 independent experiments. *n* = 3. Data are expressed as mean ± SD. Statistical analysis was performed using 2-tailed Student’s *t* test (**B**) or 2-way ANOVA with Tukey’s test for differences among groups (**E**, **F**, **H**, **I**, and **L**). *P < 0.05; **P < 0.01.

Figure 7A shows a schematic of the workflow of the animal experiments. To evaluate whether H3K18la inhibition attenuated neuronal damage in the hippocampus, we reduced microglia histone Kla levels with adeno-associated viral HDAC3-EYFP (AAV-HDAC3-EYFP) (Figure 7, B and C). The AAV infection efficiency was higher than 70% in Iba1-positive cells (Supplemental Figure 7, A and B). HDAC3 is a classical deacetylase for histone H3 Kla. In the novel object location recognition (NOL) test, control mice spent more time exploring the object in the new location (Figure 7, D–F). Similarly, mice in the T2DM+AAV-HDAC3-EYFP group also showed a significant increase in time spent interacting with the object at the novel location. However, the T2DM+AAV-EYFP mice displayed no preference between the 2 objects. Both the NOL and novel object recognition (NOR) tests indicated that AAV-HDAC3-EYFP helped alleviate cognitive impairment in T2DM mice. Spatial learning and memory were further evaluated using the Morris water maze (Figure 7G). T2DM mice took significantly longer to find the hidden platform compared with age-matched controls (Figure 7H). AAV-HDAC3-EYFP improved learning performance, as reflected by shorter escape latency during the place navigation task. In the subsequent probe trials, T2DM+AAV-EYFP mice showed less preference for the trained target zone compared with the control group. In contrast, mice in the T2DM+AAV-HDAC3-EYFP group spent significantly more time in the target quadrant compared with the T2DM+AAV-EYFP group (Figure 7I). H&E staining further confirmed that inhibition of histone Kla improved hippocampal damage (Figure 7J). TUNEL assay demonstrated that TUNEL-positive cells were increased in T2DM hippocampal slices, and were decreased after AAV-HDAC3-EYFP intervention (Supplemental Figure 7, C and D). More dead cells were observed in the hippocampus of T2DM mice. The dead cells were markedly reduced after the AAV-HDAC3-EYFP was administered (Supplemental Figure 7, E and F). In the gliosis detection experiment, compared with cells in the Control+AAV-EYFP group, GFAP fluorescence intensity increased markedly in both astrocytes and microglia of the T2DM+AAV-EYFP group (Supplemental Figure 7, G and H). However, the increased fluorescence intensity of GFAP in the T2DM+AAV-EYFP group was significantly reduced after the AAV-HDAC3-EYFP intervention. In addition, our study demonstrated that T2DM increased the expression of H3K18la and the TLR4/NF-κB pathway. However, the suppression of Kla failed to initiate TLR4/NF-κB pathway activation (Figure 7, K and L, and Supplemental Figure 2, C–F). Immunofluorescence experiments also showed that H3K18la and TLR4 were coexpressed (Supplemental Figure 8, A and B). The fluorescence intensity of H3K18la and TLR4 in T2DM microglia increased significantly. However, after treatment with AAV-HDAC3-EYFP, this fluorescence was effectively inhibited.

### Suppression of H3K18la inhibited TLR4 signaling and reduced M1 polarization.

**Figure 8:**
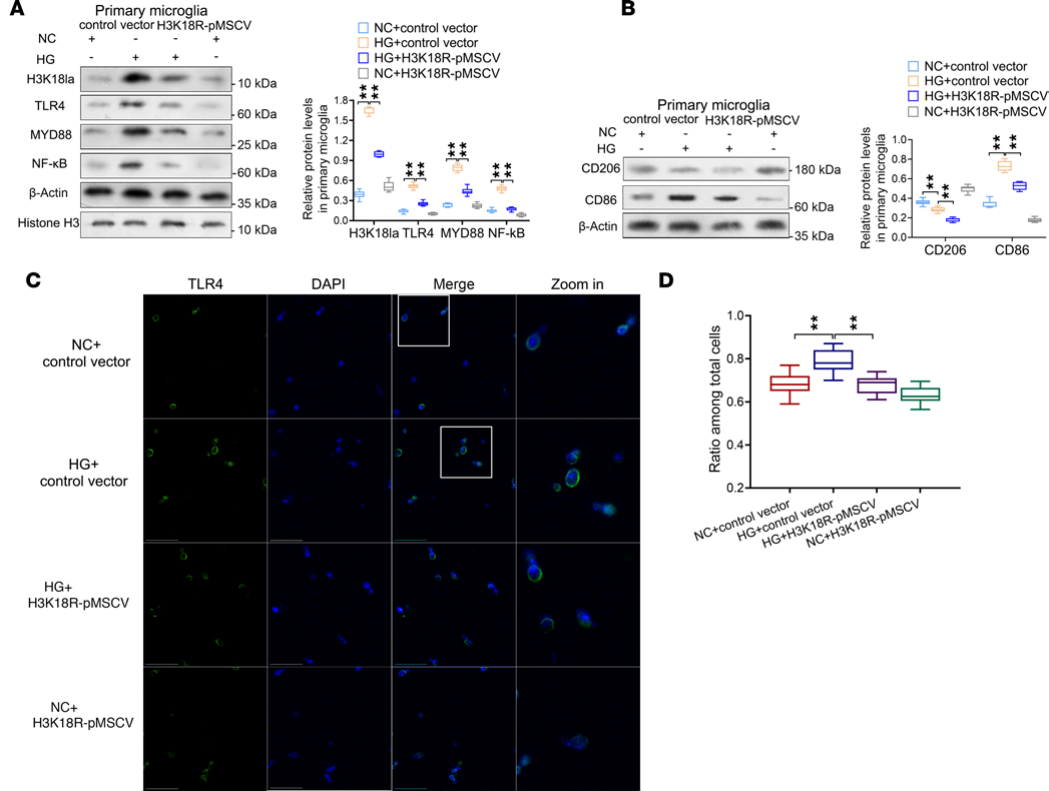
Inhibition of Kla of histone H3 at lysine 18 leads to TLR4/NF-κB suppression and microglial M1 polarization inhibition in vitro. (**A**) Western blotting analysis of TLR4/NF-κB signaling in primary microglia; graphs are representative of 3 independent experiments. *n* = 3. (**B**) Western blotting analysis of M1/M2 polarization markers in primary microglia. *n* = 3. (**C**) The representative images of immunofluorescent foci of TLR4 (green) in hippocampal tissues from 2 groups of mice (scale bars: 20 μm). (**D**) Quantitation of immunofluorescence for TLR4 in microglia. *n* = 3 per group. Data are expressed as mean ± SD. Statistical analysis was performed using 2-way ANOVA with Tukey’s test for differences among groups (**A**, **B**, and **D**). **P < 0.01.

To examine the influence of H3K18la on TLR4 signaling, we treated primary microglial cells with HG, H3K18R-pMSCV, or Control vector and then analyzed the results. First, a cell viability assay indicated that exposure to siP300 did not significantly impact cell viability (Supplemental Figure 3G). Western blot assays confirmed that H3K18la was substantially suppressed by H3K18R-pMSCV (Figure 8A). The TLR4/NF-κB pathway was activated, and microglial M1 polarization was enhanced after incubating primary microglia and HMC3 cells with 25 μM NaLa (Supplemental Figure 5, B and D). HG+Control vector treatment increased the levels of H3K18la, TLR4, MYD88, and NF-κB in primary microglial cells compared with those in NC+Control vector group. Concurrent treatment with H3K18R-pMSCV reversed the TLR4/NF-κB pathway activation (Figure 8A and Supplemental Figure 9, A and B). Immunofluorescent staining revealed the localization of TLR4 (green fluorescence) in the primary microglias treated with HG+Control vector (Figure 8C). The fluorescence intensity of the HG+Control vector group was much stronger than that of the NC group; nevertheless, the fluorescence intensity of the HG+H3K18R-pMSCV group decreased significantly (Figure 8D).

Next, we investigated the impact of the H3K18la/TLR4 signaling axis on microglial polarization by treating primary microglias with or without HG, H3K18R-pMSCV, or Control vector. Western blot data revealed that compared with the NC group, HG-treated primary microglia exhibited a significant increase in M1 polarization markers, including CD86 and iNOS. However, following the addition of H3K18R-pMSCV to HG medium, the expression of CD86 was attenuated (Figure 8B and Supplemental Figure 9, C and D). This finding suggested that the increase in M1 polarization markers was triggered by HG-induced H3K18la/TLR4 signaling activation. Immunofluorescence assays showed that HG induced decreased expression of CD206 and increased expression of CD86. Suppression of H3K18la further inhibited CD86 expression in HG primary microglia (Supplemental Figure 10A). With the addition of H3K18R-pMSCV, suppression of H3K18la resulted CD86 inhibition in primary microglia (Supplemental Figure 10B). This revealed that H3K18R-pMSCV blocked the activation of TLR4 as well as the transformation of M2-type microglia to M1-type microglia.

## Discussion

A common comorbidity of diabetes is cognitive deterioration (16–18), characterized by a marked increase in the risk of mild cognitive impairment to full-scale dementia among diabetic patients relative to healthy individuals (19). There are 3 characteristic stages in the clinical course of DACD: diabetes-linked cognitive dysfunction, mild neurocognitive impairment, and severe cognitive loss (20). However, the complexity of its pathogenesis involves the interplay of multiple factors and is yet to be fully deciphered (21, 22). DACD is considered to be a clinical outcome mediated by the chronic adverse influence of DM on cerebral function, predominantly stemming from energy metabolic disturbances (23, 24). There are many possible mechanisms of DACD (25–30), but the exact mechanism is not clear. A recent study demonstrated that lactate, which serves as both an end-product of glycolysis and an important metabolic fuel, influences transcriptional activity via the Kla of histones, depending on changes in cellular lactate availability. This discovery highlights lactate as a novel posttranslational modification contributor to the epigenetic landscape (31). Histone Kla, akin to other forms of histone acylation, has been demonstrated to directly promote transcriptional activity within chromatin contexts. Histone Kla serves an essential function in numerous biological processes, such as macrophage M1 polarization and cellular senescence. It has been linked to the underlying mechanisms of several pathological states, including the advancement of AD progression (31, 32). Studies conducted in HG, medium-glucose, and hyperglycemic animal models have shown elevated levels of L-lactate in the brains of individuals with diabetes and AD. Additionally, excessive lactate secretion in the hippocampus has been found to have an inhibitory effect on cognitive function (3, 33, 34). As the disease progressed, the T2DM mice in the present study exhibited cognitive impairments, particularly in terms of memory decline and reduced learning ability. This study revealed elevated lactate concentrations in the hippocampal and cortical regions of DACD mice, indicating a metabolic reprogramming from OXPHOS toward aerobic glycolysis. This shift facilitates the rapid generation of 5′-triphosphate (ATP) while concurrently promoting substantial lactate buildup. Furthermore, the accumulation of lactate triggered by HG leads to heightened levels of histone Kla, thereby exacerbating cognitive impairment development and Aβ deposition. These initial findings imply that the metabolic reprogramming to aerobic glycolysis within the brains of T2DM mice may have marked effects on both the hippocampus and cortex, particularly changes in microglial activity.

Next, we performed Kla proteomics analysis to gain mechanistic insight into hyperglycemia-induced microglial damage. A marked elevation in histone Kla was observed in the microglia of T2DM mice compared with control animals. This finding is similar to the results of proteomic analysis of histone Kla in senescent primary microglia (32). Protein Kla modifications include histone Kla and nonhistone Kla. Among these epigenetic marks, histone Kla serves as a key regulator of gene transcription (35). In eukaryotic cells, histones are nuclear proteins that are essential for packaging and condensing DNA into chromatin structures (36–38). Histones are composed of small, positively charged proteins that bind tightly to the negatively charged phosphate backbone of DNA. The 5 major histone families include H1, H2A, H2B, H3, and H4 (39). A key function of histones is to assemble DNA into structural subunits called nucleosomes, which constitute the fundamental repeating units of chromatin and are formed by an octamer of histone proteins (2 each of H2A, H2B, H3, and H4) encircled by approximately 147 DNA base pairs. The linker histone H1 binds to DNA between nucleosomes, promoting higher-order chromatin organization (40, 41). This packaging allows DNA to be tightly compacted, regulating access to genetic information and influencing processes such as transcription, replication, and DNA repair (35).

We observed significant differences in H3K18la between microglia in T2DM mice and control mice. H3K18la has been shown to be critically involved in multiple pathological contexts, such cancer, cardiovascular diseases, and metabolic diseases (32, 42, 43). This modification, which involves the attachment of lactyl moieties to lysine 18 on histone H3, regulates chromatin structure and gene expression, thus impacting cellular functions and disease progression. Dysregulation of H3K18la has been implicated in aberrant transcriptional programs, disrupted DNA repair mechanisms, and altered signaling pathways, all of which contribute to disease pathogenesis. Understanding the precise mechanisms underlying H3K18la in different pathological contexts may provide a foundation for novel therapeutic approaches. GO analysis identified genes associated with key ontological categories, encompassing biological processes, cellular components, and molecular functions. Notably, the most enriched terms included regulation of biological processes, cellular metabolic processes, protein binding, and DNA-binding transcription factor activity. In addition, we observed strong enrichment of TLR signaling pathway members in the gene set enrichment analysis.

Through CUT&Tag profiling and subsequent validation experiments, we demonstrated that H3K18la stimulates the TLR4/NF-κB pathway to promote M1 polarization. This finding suggests that transition to aerobic glycolysis in HG-stimulated microglia exacerbates DACD via neuroinflammatory mechanisms mediated by the H3K18la/TLR4 signaling axis. This findings illuminate a prospective biological implication of histone Kla and its regulatory influence on downstream genes or signaling cascades. These data advance the current knowledge of the functional scope of histone Kla and its target networks. Initial evidence suggest that the H3K18la/TLR4/NF-κB axis exacerbates DACD phenotypic progression by amplifying neuroinflammatory responses in microglia under hyperglycemic conditions. Collectively, the aforementioned findings imply that the effects of the Kla of various histones are exerted via diverse molecular mechanisms, influencing specific target genes or pathways in microglia and thereby promoting cerebral impairment and cognitive dysfunction. Subsequent experimental validation showed that increased H3K18la result in heightened binding to the promoter region of the TLR4 gene, consequently amplifying NF-κB signaling and ultimately facilitating microglial M1 polarization. These effects substantially influence the inflammatory status of the microglial cells. Our research indicates that H3K18la/TLR4/M1-M2 polarization promotes the pathogenesis of DACD. Additionally, we demonstrated the acetyltransferases P300 and CBP function as histone lactyltransferases (designated as “writers”) in 2 distinct microglial systems: primary cultures and the HMC3 cell line. This finding shows partial consistency with previous studies by Zhang et al. (13) and Wei et al. (32).

The present study possesses certain limitations. First, our study did not establish animal models with site mutations of histone H3. The lactoyl group’s unique chemical structure cannot be directly mimicked through conventional amino acid substitutions. The absence of documented CRISPR/dCas9 applications for site-specific Kla control reflects an inevitable methodological gap. Compounding this limitation, current epigenetic editing tools universally lack the capacity to induce gene repression magnitudes comparable to native epigenetic regulation (44). There has been no reliable animal model with these particular site mutations available to test the effect on TLR4 and M1 polarization in vivo. Forthcoming research aimed at delineating the specific actyltransferases or delactylases, as well as the proteins responsible for writing, erasing, and/or interpreting H3K18la, will greatly advance our understanding of how this epigenetic mark influences microglia in T2DM. Furthermore, elucidating the mechanistic conservation and disease-driving functions of this axis in additional microglia-driven neuroinflammatory disorders, such as Parkinson disease, ischemic stroke, and multiple sclerosis, would be of considerable interest.

**Figure 9:**
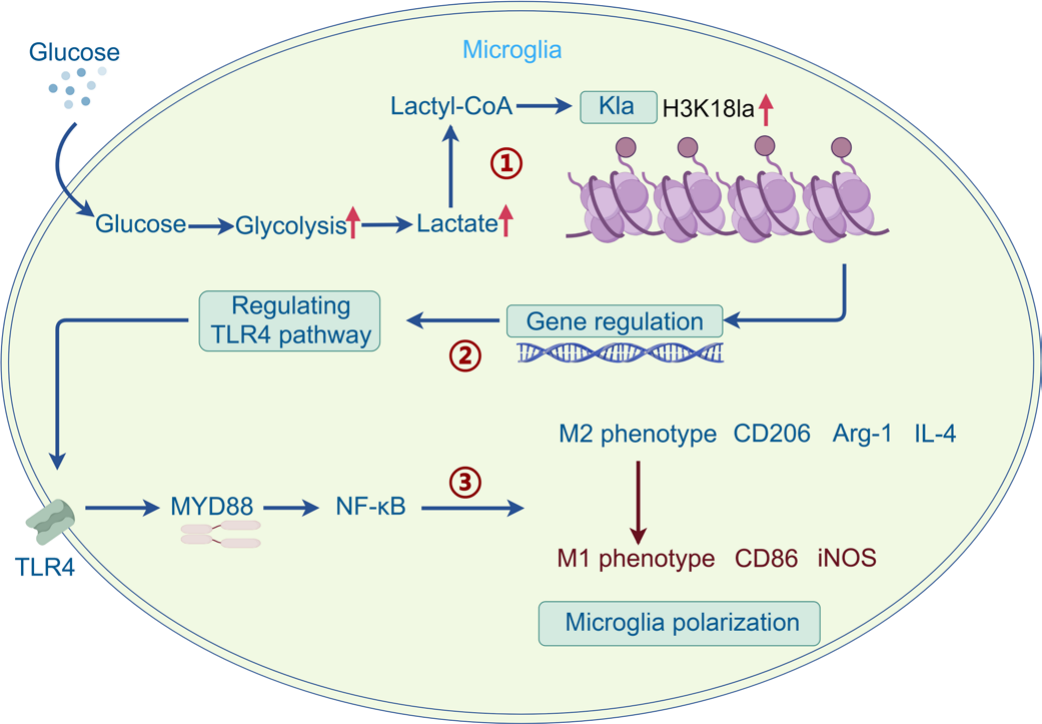
Mechanism diagram.

In conclusion, this study unveil a previously unrecognized mechanism through which the H3K18la/TLR4 axis orchestrates neuroinflammatory responses by influencing microglial polarization, thereby fostering the emergence of DACD phenotypes (refer to Figure 9). These findings highlight therapeutic targets for the advancement of promising interventions aimed at addressing DACD pathology.

## Methods

### Sex as a biological variable.

For experiments in animals, only male mice were used based on practical considerations. This approach reduces variability from the female estrous cycle, allowing us to focus resources on delineating the core mechanism.

### Animal care and ethics statement.

Eight-week-old male C57BL/6J mice were procured from Beijing SiPeiFu Biotechnology Co., Ltd. [SCXK (Jing) 2019-0004]. Mice were group-housed with 4–5 animals per cage under standard temperature conditions and a 12-hour light/dark cycle. The mice had ad libitum access to food and water throughout the experiments. The T2DM mouse model was induced following a previously described protocol (45). Briefly, mice were fed an HFD containing 60% fat for 4 weeks prior to i.p. injection of STZ at a dosage of 25 mg/kg/day for 5 consecutive days. Control mice received an equivalent volume of phosphate-buffered saline (PBS, 1×) i.p. for 5 consecutive days after being fed a standard chow diet for 4 weeks. Diabetes was confirmed if blood glucose levels exceeded 300 mg/dL 16 days after the last STZ injection. Blood glucose levels were measured from tail vein blood samples using ACCU-CHEK glucose strips. The weights were recorded and circulating triglycerides and free fatty acids were measured with commercial kits (TR0100, Sigma-Aldrich).

As for lateral ventricle injection (i.c.v.) with AAVs, mice were anesthetized by i.p. injection of pentobarbital, and the head was fixed in a stereotactic frame. Injections were administered into each lateral ventricle using 0.5 μL of viral preparation, delivered through a needle connected to a 10-μL Hamilton syringe (Hamilton Medical) at a rate of 0.25 μL per minute. Stereotactic coordinates were calculated from bregma (anteroposterior –2.2 mm, mediolateral ± 1.5 mm, and dorsoventral –1.5 mm).

### Morris water maze test.

The Morris water maze test was conducted to assess spatial learning and memory capacity. The study protocol has been described previously (46). The test was conducted 16 weeks after model establishment. The test utilized a gray circular pool with a diameter of 150 cm, divided into 4 equal quadrants, each filled with water maintained at 30°C ± 2°C and with a depth of 16 cm. An overhead camera was positioned centrally above the pool and linked to a computerized recording system equipped with a tracking program (DigBehv, Shanghai Jiliang Software Technology Co., Ltd.) capable of monitoring and recording the swimming paths of the mice.

The testing protocol consisted of 4 platform trials per day over 5 consecutive days (acquisition phase), followed by a probe trial on the sixth day. During the acquisition phase, the mice were introduced into the water pool within a quadrant devoid of the platform and given 60 seconds to locate the hidden platform. If a mouse failed to locate the platform within the allotted time, it was gently guided to the platform and allowed to remain there for 15 seconds to reinforce the memory of its location. The measurements were recorded at 90 seconds. This procedure was repeated 4 times daily until no significant improvement in performance was observed. On the sixth day, the platform was removed, and each mouse was permitted to swim freely for 60 seconds from the same starting position, opposite to the original platform location. The time spent in the target quadrant and total swimming duration were recorded for analysis.

### NOR and NOL tests.

NOR and NOL tests were conducted to evaluate learning and memory abilities in each group. These assessments were performed 16 weeks after the experimental model was established. To begin, the mice were acclimated to an empty wooden chamber for 15 minutes. After a 24-hour interval, each mouse was reintroduced to the chamber, now containing 2 identical objects positioned in opposite corners. The mice were allowed to freely explore the area until they had spent a combined 30 seconds interacting with both objects. Interaction was defined as the mouse approaching an object with its nose within 2 cm of it; sitting or climbing on the object was not considered exploration.

After another 24 hours, the mice were returned to the chamber where one of the familiar objects was replaced with a novel object placed in a different corner. The time spent exploring the new object, relative to the total exploration time for both objects, was recorded to determine the preference for novelty. Eight mice from each group were randomly selected for these tests. The NOL test was carried out first, followed by the NOR test 4 days later. In the NOR test, 24 hours after the initial exploration phase, one of the familiar objects was replaced with a new one, and the proportion of time spent exploring the novel object was calculated in relation to the total exploration time for both objects.

### H&E staining.

At 16 weeks after model establishment, the mice were anesthetized by i.p. injection of pentobarbital, followed by transcardial perfusion with 20 mL of ice-cold PBS. To assess neuronal damage, H&E staining was performed following the manufacturer’s protocol from Solarbio Science and Technology in Beijing, China. Histological changes in the hippocampal regions were observed under an optical microscope (Olympus Corporation; ×400 magnification). The quantification of neurons within the CA1, CA3, dentate gyrus pyramidal cell layer, and cortex was conducted by counting the number of neurons per 250-μm length in 5 sections per mouse, with the average serving as the final outcome.

### TUNEL staining.

Apoptosis was assessed via the TUNEL assay using a commercial kit (C1088, Beyotime) following standardized protocols. Briefly, fixed tissue sections or cellular specimens were permeabilized with 0.5% Triton X-100/PBS (5 minutes, room temperature), then incubated with TUNEL reaction mixture at 37°C for 60 minutes under light-protected and evaporation-controlled conditions. Nuclei were counterstained with 4′,6-diamidino-2-phenylindole (DAPI, 10 minutes), followed by 3 PBS washes (5 minutes each). Slides were mounted with 50% glycerol–based medium and imaged under a fluorescence microscope (Olympus) at ×400 magnification. Neuronal apoptosis rates were quantified by analyzing 5 randomized fields, calculating the percentage of TUNEL-positive cells relative to DAPI-stained total neurons as (TUNEL^+^/total) × 100.

### Lactate measurement.

Serum collected from living mice was collected for lactate measurements. To minimize postmortem metabolic changes, after anesthetizing the mice, brains were fixed via microwave irradiation (>5 kW; 2.45 GHz). Brain tissue was then rapidly excised and immediately flash-frozen in liquid nitrogen. The frozen hippocampal tissue was homogenized with 1 mL of lactate assay buffer on ice, followed by centrifugation at 12,000*g* for 5 minutes at 4°C. The resulting supernatant was utilized for the quantification of lactate levels using an L-lactate assay kit (A019-2-1, Jiancheng) according to the manufacturer’s instructions. For in vitro experiments involving microglia, the cells were harvested, transferred to centrifuge tubes, and washed with precooled PBS. Following centrifugation, the supernatant was decanted and subsequently supplemented with 1 mL of lactate assay buffer. Subsequently, the cells were sonicated via ultrasonication in an ice bath for 5 minutes at a power of 200 W with intervals of 3 seconds on and 7 seconds off; this process was repeated 30 times. The resulting mixture was then subjected to a second centrifugation at 12,000*g* for 5 minutes at 4°C, after which the resulting supernatant was carefully transferred to a fresh EP tube. Lactate levels within brain tissues and microglia in vitro were quantified utilizing a lactate analyzer (Roche).

### Live cell staining.

For the in vivo experiments, fresh hippocampal slices were stained with calcein-AM to visualize intracellular calcium activity (indicating live cells) and ethidium homodimer-1 (marking dead cells), employing the Live/Dead Cell Viability Assay Kit (Invitrogen) according to the manufacturer’s protocol. Following staining, slices underwent 3 washes with DPBS and were subsequently fixed using 3.7% paraformaldehyde. Fixed slices were then counterstained with DAPI diluted 1:400 in DPBS. Fluorescence was assessed using a fluorescence microscope with excitation/emission wavelengths of 494 nm and 528 nm, respectively.

### Immunofluorescent and immunohistochemical staining.

Sections were deparaffinized and rehydrated prior to immunofluorescent staining. Sections or cells were blocked with goat serum for 1 hour, incubated at 4°C overnight with primary antibodies against Iba1 (catalog MA5-50414; 1:500 dilution; RRID: AB_3094208, Invitrogen/Thermo Fisher Scientific), Pan-Kla (catalog PTM-1401RM; 1:100 dilution; RRID:AB_2942013, PTM BIO), H3K18la (catalog PTM-1406RM; 1:100 dilution; RRID:AB_3076698, PTM BIO), Aβ (catalog MA1-140; 1:500 dilution; RRID: AB_2536844, Invitrogen/Thermo Fisher Scientific), TLR4 (catalog MA5-16216; 1:500 dilution; RRID: AB_2537734, Invitrogen/Thermo Fisher Scientific), CD86 (catalog 942-MSM1-P0; 1:500 dilution; RRID: AB_3101867, NeoBiotechnologies), CD206 (catalog PA5-101657; 1:500 dilution; Invitrogen/Thermo Fisher Scientific), and iNOS (catalog PA1-036; 1:500 dilution; RRID: AB_325773, Invitrogen/Thermo Fisher Scientific); washed; incubated with secondary antibodies (Alexa Fluor 488 conjugated [green], Alexa Fluor 594 conjugated [red], Alexa Fluor 750 conjugated [pink], Invitrogen/Thermo Fisher Scientific); and then mounted on slides. The mounted slides were examined and captured with an Axio Observer 3 fluorescence microscope (Zeiss). ImageJ software (NIH) was employed to gauge the mean fluorescence intensity, while Iba1-positive and Kla-positive cells were manually enumerated. Since GFAP constitutes the main intermediate filament protein in glial cells, reactive gliosis can be assessed via GFAP immunostaining. The primary antibody for GFAP was rabbit polyclonal anti-GFAP antibody (catalog 345860, Millipore). To assess the transcriptional states of sorted microglial cells, we quantified fluorescence intensity of key phenotypic markers: homeostatic markers (TMEM119, P2RY12) and activation markers (CD68, IL-1β). The antibody information is as follows: TMEM119 (catalog PA5-119902; 1:500 dilution; RRID: AB_2913474, Invitrogen/Thermo Fisher Scientific), P2RY12 (catalog 702516; 1:500 dilution; RRID: AB_2689476, Invitrogen/Thermo Fisher Scientific), CD68 (catalog MA5-56511; 1:500 dilution; RRID: AB_3679538, Invitrogen/Thermo Fisher Scientific), and IL-1β (catalog P420B; 1:500 dilution; RRID: AB_223478, Invitrogen/Thermo Fisher Scientific).

The paraffin blocks were cut into 4- to 5-μm sections for immunohistochemical staining. The experimental protocols were performed following the instructions outlined in the DAB kit and the IHC kit (ZSGB-BIO). Subsequently, the slides were mounted, examined, and photographed using an Olympus BX53 microscope.

### Isolation of microglia from adult mouse brains.

Following brain extraction, the cortices, olfactory bulbs, and cerebellum were removed, and the bilateral hippocampi were harvested. To avoid microglial polarization, cell sorting was conducted in a brief period of controlled hypothermia after gentle dissociation with the Adult Brain Dissociation Kit (130-107-677, Miltenyi Biotec) following the manufacturer’s instructions. After myelin removal with Cell Debris removal buffers, the total cell pellet was resuspended in PBS containing 0.5% BSA for specific cell type isolation. Microglia were isolated using anti-CD11b–coated MicroBeads (130-093-634, Miltenyi Biotec) with a MACS multistand separator according to the manufacturer’s instructions. For histone extraction and examination, isolated cells were pooled from 3 mice for each sample. After confirming by immunofluorescence, sorted cells did not polarize and with no or very few cellular protrusions (Supplemental Figure 1, A and B).

### Trypan blue exclusion test.

The trypan blue exclusion method served as the standard viability assessment. After 0.2% trypan blue staining, cell counts (viable vs. non-viable) were obtained in triplicate via a Countstar BioTech Automated Cell Counter (Alit Life Science), evaluating 8 chamber squares per replicate. Viable cell percentages were calculated (Supplemental Figure 1C). A minimum of 1 × 10^7^ cells were processed for subsequent experiments.

### Flow cytometry.

Microglial purity was determined via flow cytometry (CytoFLEX LX, Beckman Coulter). Isolated cells were immunophenotyped as CD11b^+^CD45^lo^ microglia, with data analyzed using FlowJo software. This rigorous quality control confirmed 98% or greater purity (Supplemental Figure 1D).

### Kla proteomics analysis.

Kla proteomics analysis was conducted by Jingjie PTM BioLabs. The procedure commenced with protein extraction from cellular samples utilizing a high-intensity ultrasonic processor sourced from Scientz. The extraction process involved a lysis buffer comprising 8 M urea and 1% protease inhibitor cocktail, followed by trypsin digestion. The resulting tryptic peptides were then tagged with the respective TMT reagents obtained from Thermo Fisher Scientific. To optimize peptide modification, the peptides were dissolved in NETN buffer (100 mM NaCl, 1 mM EDTA, 50 mM Tris-HCl, 0.5% NP-40, pH 8.0) and incubated overnight at 4°C with gently agitated prewashed antibody beads sourced from Jingjie PTM BioLabs. Subsequent to this step, the peptides were desalted utilizing C18 ZipTips from Millipore, to prepare for liquid chromatography coupled with tandem mass spectrometry (LC-MS/MS) analysis. These tryptic peptides were then introduced to a tailor-made reversed-phase analytical column (25 cm length, 75 μm i.d.) and separated using an EASY-nLC 1200 UPLC system procured from Thermo Fisher Scientific. The isolated peptides were analyzed with a Q ExactiveTM HF-X mass spectrometer (Thermo Fisher Scientific) equipped with a nanoelectrospray ion source. The resulting MS/MS data were processed via the MaxQuant search engine (v.1.6.15.0). Furthermore, the study included GO annotation, domain annotation, subcellular localization analysis, KEGG pathway enrichment, and clusters of orthologous groups (COG)/eukaryotic orthologous groups (KOG) functional classification.

### Western blot analysis.

After quantifying the total protein concentration extracted from microglia both in vitro and in vivo, a loading buffer was introduced for high-temperature denaturation. Following this step, the protein samples underwent separation via SDS-PAGE (Wako Supersep Ace; 5%–20%) and were subsequently transferred onto PVDF membranes (Invitrogen) immersed in a transfer buffer. To prevent nonspecific binding, the blots were treated with 5% nonfat blocking-grade milk for blocking purposes. Primary antibodies against Pan-Kla (RRID:AB_2942013, Jingjie PTM BioLabs, PTM-1401RM), histone H3K18 (RRID:AB_3076698, Jingjie PTM BioLabs, PTM-1427RM), histone H4K12 (RRID:AB_2941896, Jingjie PTM BioLabs, PTM-1411RM), histone H3K23 (RRID: AB_3101865, Jingjie PTM BioLabs, PTM-1413RM), histone H3 (Jingjie PTM BioLabs, PTM-1001RM), histone H4 (RRID: AB_3101866, Jingjie PTM BioLabs, PTM-1015RM), TLR4, NF-κB, CD86, CD206 (RRID: AB_2537734, RRID AB_2533893, RRID: AB_3101867, RRID: AB_2851091, all from Invitrogen/Thermo Fisher Scientific, MA5-16216, 51-0500, 942-MSM2-P0, PA5-101657) were applied to the blots overnight. Following washing with Tris-buffered saline containing 0.05% Tween 20 (TBST), the blots were incubated with a secondary antibody (Cell Signalling Technology, CST-7074S) at room temperature for 1 hour. Detection of specific antibody fluorescence density of the protein bands was achieved using the enhanced chemiluminescent reagent (Bio-Rad Laboratories, Inc.). Blots were repeated 3 times independently, with 3 technical repeats. The relative density of each band was quantified using ImageJ (NIH version 1.0) and indicated under each immunoblot after normalization to density of the β-actin band in the corresponding lane. Uncropped blots are shown in the supplemental material.

### qPCR.

Tissues or cells were lysed using a TRIzol-based cell lysis buffer (Invitrogen). Subsequently, cDNA was synthesized using a PrimeScript RT reagent kit (RR037A, Takara Bio, Inc.), followed by qPCR analysis using SYBR Green mix (Roche) to quantify gene expression levels. The primer sequences are listed in Supplemental Table 1. β-Actin was employed as the reference gene for normalization of mRNA expression levels.

### Cell culture.

Primary microglia cultures were prepared from C57BL/6J mice using established protocols (47). The cortices of 2-day-old mice were minced in Hibernate A medium containing B27 (Gibco) and dissociated using a papain (Worthington) solution. Following tissue trituration, cells were isolated by Optiprep (Sigma-Aldrich) density gradient centrifugation. The microglia were then seeded in a microglia-specific medium consisting of DMEM/F12 supplemented with 10% FBS and 1% penicillin-streptomycin. HMC3 cells (CVCL_II76) were cultured in minimum essential medium (MEM) supplemented with non-essential amino acids (NEAA) and 10% FBS. Both cell types were passaged every 3–4 days and maintained in a constant-temperature incubator at 37°C. Microglia were seeded at a density of 1 × 10^6^ cells per well in a 6-well plate. Cells were cultured with or without NaLa (25 μM; >99% pure, Sigma-Aldrich) and incubated for 24 hours. To mimic the HFD in vivo model of diabetes, we used HG (25 mM glucose) in in vitro experiments. We also set the concentrations of moderate glucose (5 mM) and low glucose (1 mM) to explore the effects of different glucose concentrations on cells. For inhibiting LDH activity, oxamate (50 mM) was added and cells incubated for 2 hours prior to the next step.

### Plasmids and siRNA.

Microglia were plated at 2 × 10^6^ cells/well in 6-well plates 1 day before transfection, and the cell monolayer reached the desired 70%–80% confluence. H3K18R-pMSCV vectors were constructed as previously described (48). Site-directed mutagenesis was employed to generate the H3K18R mutants. Lipofectamine 2000 reagent (Invitrogen) was used for plasmid transfection according to instructions provided by the manufacturer (Supplemental Table 2). For siRNA transfection, primary microglia and HMC3 cells were transfected at 80% confluence in 6-well plates with 40 pmol/well nontargeting control siRNA (con si) or P300-targeting siRNA (siP300) for 48 hours.

### CUT&Tag and sequencing.

The isolated microglia were promptly utilized for the CUT&Tag experiment. The CUT&Tag method was carried out using the Hieff NGS G-Type In-situ DNA Binding Profiling Library Prep Kit for Illumina (12598ES12, Yeasen) following the manufacturer’s protocol. Cells were bound to concanavalin A–coated beads, and then resuspended in antibody buffer for incubation with primary antibodies targeting H3K18la (Jingjie PTM BioLabs, PTM-1427RM), followed by incubation with secondary antibodies (Abcam, ab6702). The samples were treated with pA-Tn5 transposase for transposon activation and tagmentation, and the resulting DNA was isolated, amplified, and purified to generate the library. CUT&Tag-seq was performed on an Illumina NovaSeq (PE150) platform.

### Transcriptome analysis.

To delineate transcriptomic changes in sorted microglial cells under hyperglycemic conditions, we conducted RNA sequencing at the Novogene Bioinformatics Institute (Novogene). Three biological replicates per group underwent total RNA extraction with TRIzol (Invitrogen), followed by rigorous quality assessment on an Agilent 2100 Bioanalyzer. Only samples meeting stringent criteria (A260/A280 > 1.8; RIN > 8.0) were processed. PolyA-selected mRNA was fragmented, reverse-transcribed, and subjected to second-strand cDNA synthesis (Invitrogen). After end-repair, adenylation, and Illumina adapter ligation, libraries were sequenced on the HiSeq 2500 system. Transcriptomic reads were aligned to the *Mus* *musculus* GRCm39 reference genome employing HISAT2 v2.2.1 (https://daehwankimlab.github.io/hisat2/) with default parameters. Gene/isoform quantification was performed via StringTie v2.1.7 (https://ccb.jhu.edu/software/stringtie/) utilizing ensemble-based transcript models. Differential expression analysis implemented DESeq2 v1.40.0 (https://github.com/mikelove/DESeq2/) under the negative binomial distribution framework, with significance thresholds set at |log_2_FC| greater than 0.58 and Benjamini-Hochberg–adjusted *P* less than 0.001. These DEGs were classified and enriched using GO and KEGG pathway annotation.

### Cell viability.

The viability of primary microglia and HMC3 cells was evaluated utilizing a CCK-8 assay (Dojindo Molecular Technologies Inc.) subsequent to treatment with H3K18R-pMSCV and siP300. Assessments were performed at 0, 6, 12, 18, 24, 30, 36, 42, 48, 54, and 60 hours under standard culture conditions or in HG media.

### Free fatty acids and triglyceride detection.

The free fatty acids of mice were analyzed using a commercial kit (Free Fatty Acid Quantification Kit, Abcam) and the triglyceride levels were measured using a Triglyceride Reagent kit (Sigma-Aldrich, Missouri, USA) (Supplemental Table 3).

### Statistics.

The statistical analyses were conducted using 2-tailed, unpaired Student’s *t* test or ANOVA test with GraphPad Prism software unless otherwise specified. Quantitative data from 3 independent biological replicates are expressed as the mean ± SD. Unless stated otherwise, all experiments were carried out with 3 independent biological replicates, and a 2-sided *P* value of less than 0.05 indicates statistical significance. For all box and whisker plots, the bounds of the boxes represent interquartile range, lines within the boxes represent median, and whiskers denote maximum and minimum values.

### Study approval.

The experimental procedures and animal care protocols were approved by the Ethics Committee of China–Japan Friendship Hospital (no. zryhyy61-24-02-25).

### Data availability.

Values for all data points in graphs are reported in the Supporting Data Values file. Additional details required for data reanalysis can be obtained by contacting the corresponding author.

## Author contributions

YY and BZ conceived the study and contributed to the interpretation of the results. YY and FC performed statistical analyses and drafted the first manuscript draft. LS, FC, LY, JZ, and BZ contributed to the critical revision of the manuscript. BZ attests that all listed authors meet authorship criteria and that no others meeting the criteria have been omitted. YY and BZ have accessed and verified the underlying data. All authors had accepted responsibility for the decision to submit for publication.

## Funding support

- National High Level Hospital Clinical Research Funding (2025-NHLHCRF-JBGS-A-WZ-09)
- Capital Health Development Research Special Project (2024-1-4064)
- National Key Research and Development Program of China (2018YFC1313902)
- Medical Science Research Project of Hebei (20220214)

## Supplementary Material

Supplemental data

Unedited blot and gel images

Supporting data values

## Version 1 11/04/2025

In-Press Preview

## Version 2 12/22/2025

Electronic publication
