# Supplementary material for: H3K18 lactylation potentiates microglial polarization via the TLR4 pathway in diabetes-induced cognitive impairment
Source: JCI Insight. 2025 Nov 4;10(24):e188077. doi: 10.1172/jci.insight.188077 (PMC12890480; doi:10.1172/jci.insight.188077)
Supplement: Supplemental data [file jciinsight-10-188077-s141.pdf]

## Supplementary material

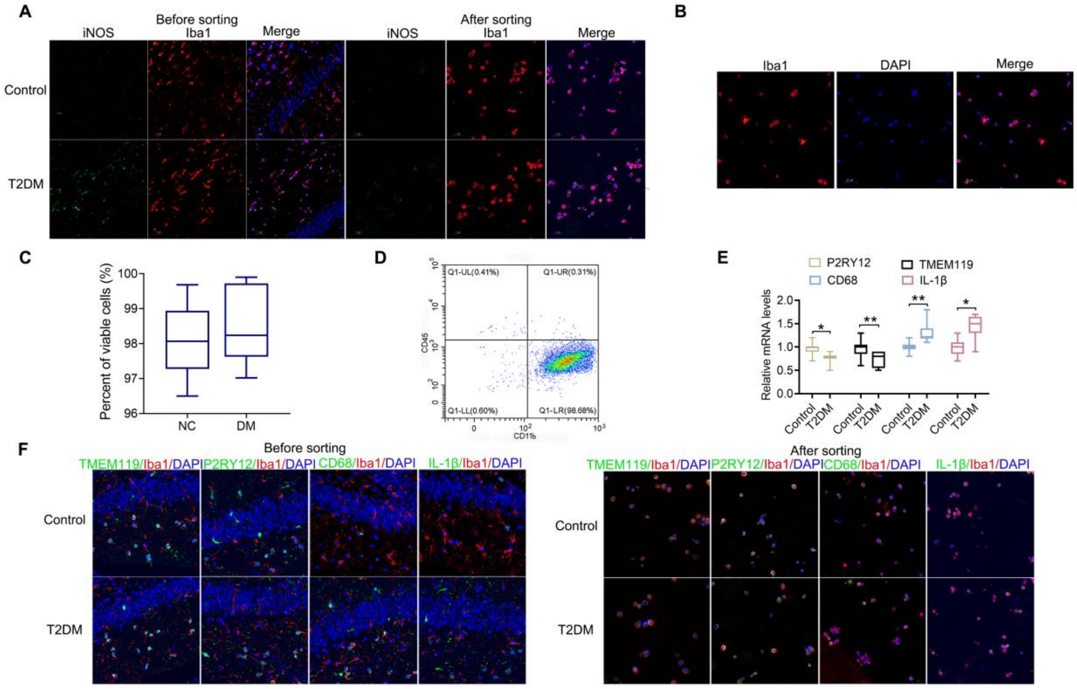


Fig.S1 Characterization of microglia before and after cell sorting. (A) Microglia phenotypes identified after sorting microglia. (B) Representative images of microglia stained for Iba1 (red) and DAPI (blue) in primary microglia. (C) Viable cell percentages were calculated after trypan blue exclusion test. (D) Purity check was performed by flow cytometry and showed purity ⩾98%. (E) mRNA expression for P2RY12, TMEM119, CD68 and IL-1β in sorted microglias. (F) Representative images of microglia stained for P2RY12, TMEM119, CD68, IL-1β (all green) and Iba1 (red) in microglias before and after sorting microglias. n=3 per group. Data are expressed as mean ± SD. Statistical analysis was performed using two-tailed Student's t tests (C, and E). $^{**}P<0.01$, $^{*}P<0.05$.

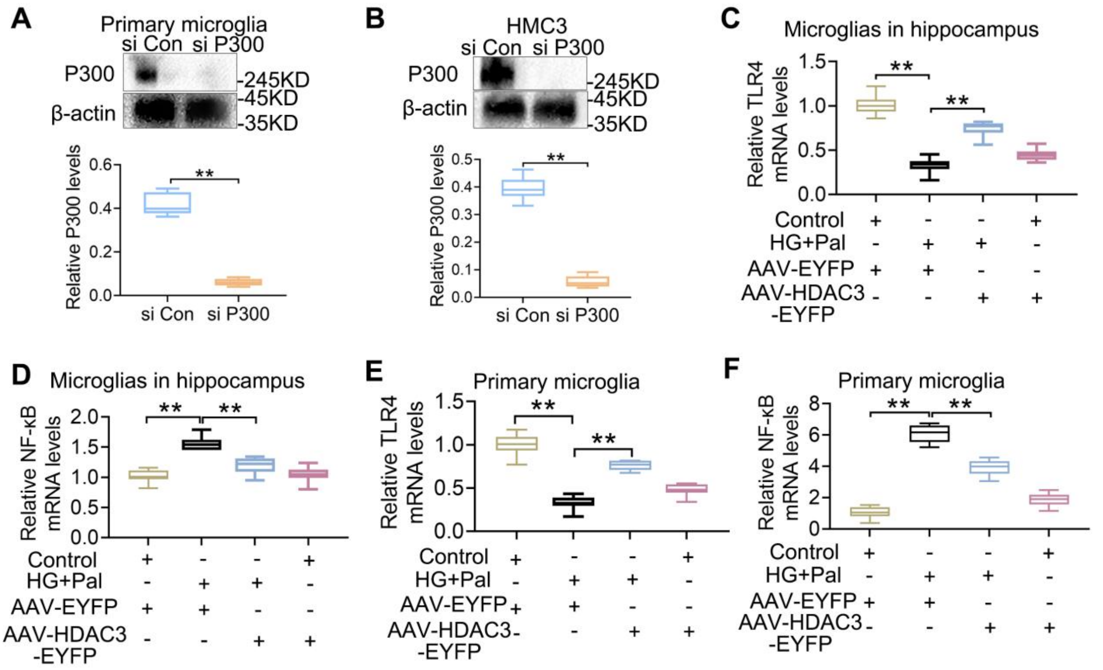


Fig.S2 Inhibition of lactylation inhibited TLR4 pathway. (A) Representative Western blotting band of P300 in primary microglias transfected with siRNA. (B) Representative Western blotting band of P300 in HMC cells transfected with siRNA. (C) mRNA expression for TLR4 in microglias in hippocampus. n=3. (D) mRNA expression for NF-κB in microglias in hippocampus. n=3. (E) mRNA expression for TLR4 in primary microglias. n=3. (F) mRNA expression for NF-κB in primary microglias. n=3 per group. Data are expressed as mean ± SD. Statistical analysis was performed using two-way ANOVA with Tukey test for differences among groups. $^{**}P<0.01$.

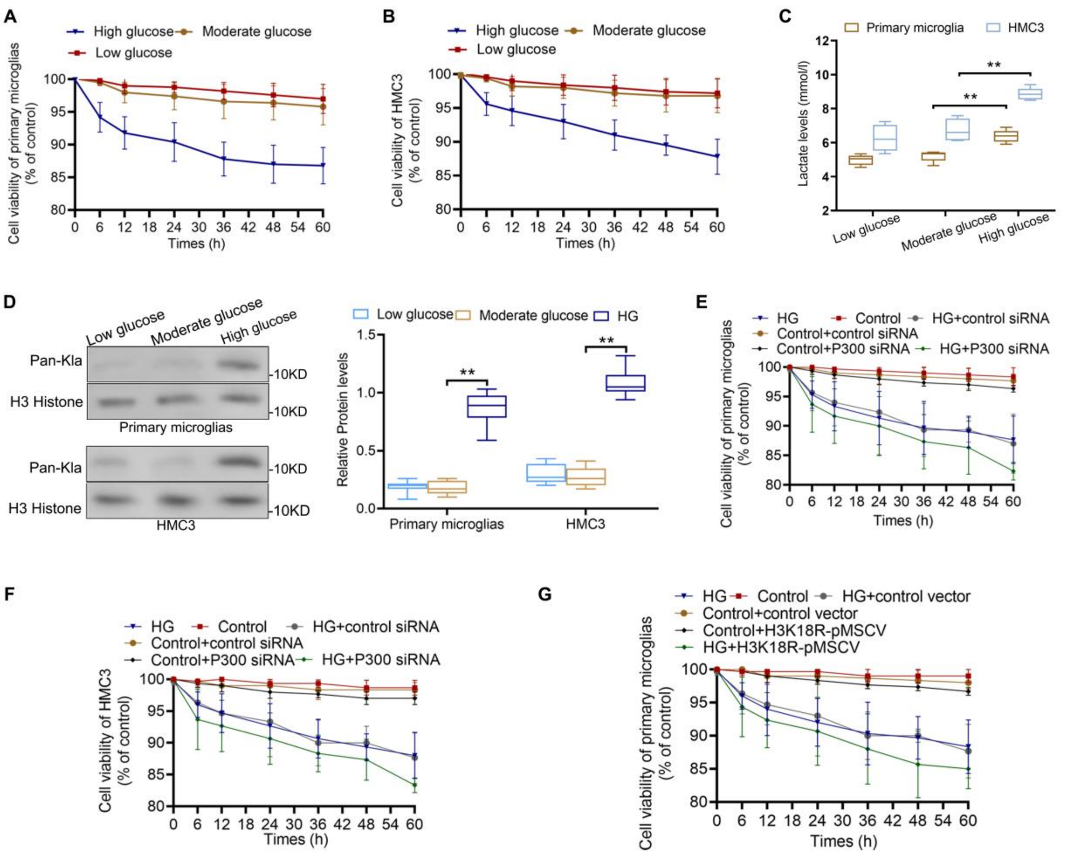


Fig. S3 The cell viability, lactate levels and Pan-Kla levels of microglias and HMC3 in different times. (A) The cell viability of primary microglias in different glucose concentration medium. (B) The cell viability of HMC3 in different glucose concentration medium. (C) The lactate levels of primary microglias and HMC3 in different glucose concentration medium when cultured until 48h. (D) Relative protein levels of Pan-Kla. (E) The cell viability of primary microglias when treated with P300 siRNA. (F) The cell viability of HMC3 when treated with P300 siRNA. (G) The cell viability of primary microglias when treated with H3K18R-pMSCV. n=3 per group. Data are expressed as mean ± SD. Statistical analysis was performed using two-tailed Student's t tests or two-way ANOVA with Tukey test for differences among groups.

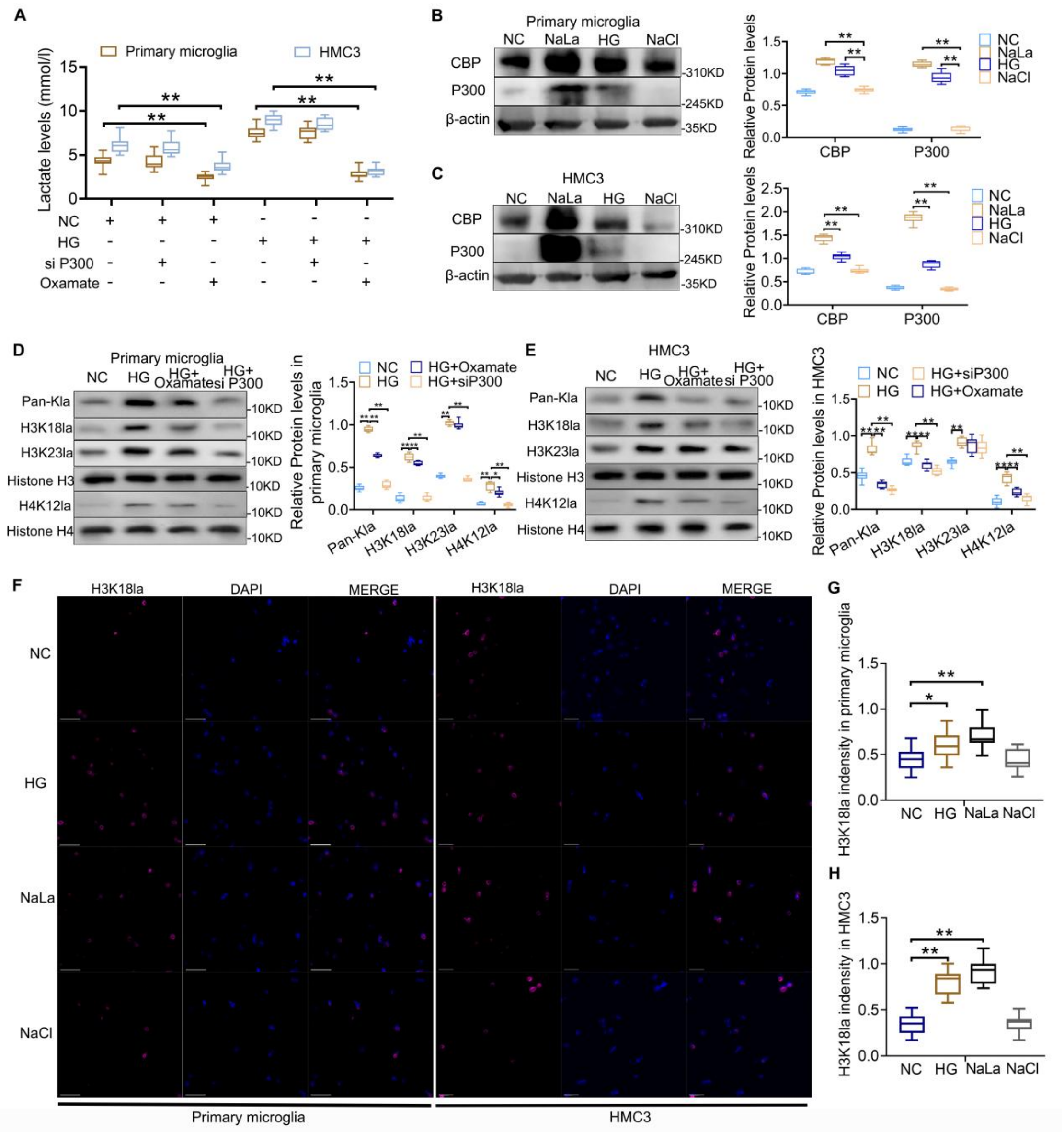


Fig.S4 Elevated levels of H3K18 lactylation can be observed in NaLa-treated microglia. (A) Lactate levels in primary microglias and HMC3 cells of different groups. n=3. Western blotting analysis of CBP and P300 in primary microglias (B) and HMC3 cells (C) of different groups. (D) Western blotting analysis of Pan-Kla, H3K18la, H3K23la and H4K12la in primary microglias and (E) HMC3 cells of different groups. (F) Fluorescence expression and localization analyses of H3K18la in the primary microglias and HMC3 cells of different groups (scale bar= 20 μm). (G) Quantification of H3K18la intensity in primary microglia. (H) Quantification of

H3K18la intensity in HMC3. More than five fields of vision were randomly selected to be analyzed. n=3 per group. Data are expressed as mean ± SD. Statistical analysis was performed using two-way ANOVA with Tukey test for differences among groups. $^{**}P<0.01$, $^{*}P<0.05$. NC, Negative control; HG, High glucose.

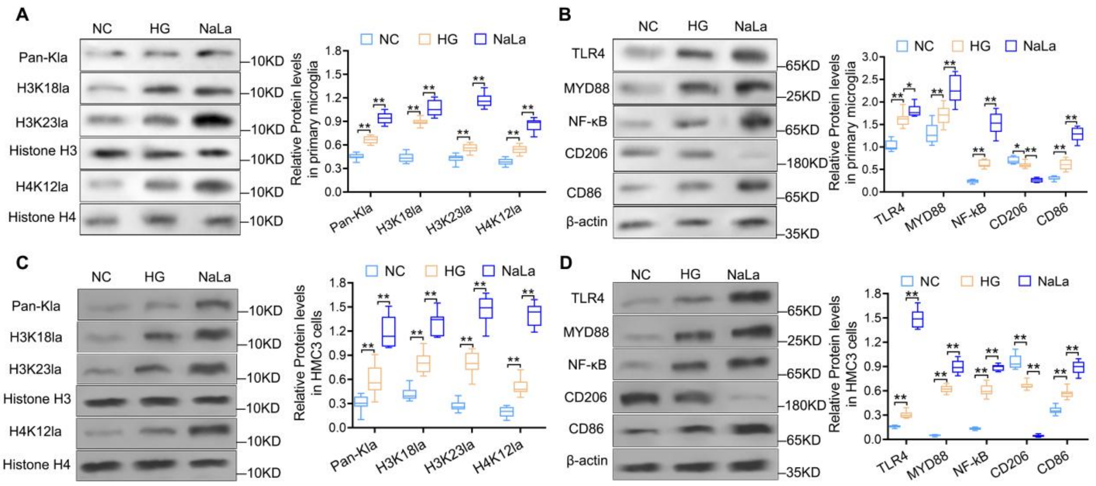


Fig.S5 The effects of NaLa incubation on histone lactylation and the TLR4/NF-κB pathway in primary microglia and HMC3 cells. (A) Effects of NaLa on histone lactylation in primary microglia. (B) Effects of NaLa on TLR4/NF-κB pathway in primary microglia. (C) Effects of NaLa on histone lactylation in HMC3 cells. (D) Effects of NaLa on TLR4/NF-κB pathway in HMC3 cells. n=3 per group. Data are expressed as mean ± SD. Statistical analysis was performed using two-way ANOVA with Tukey test for differences among groups. $^{**}P<0.01$, $^{*}P<0.05$.

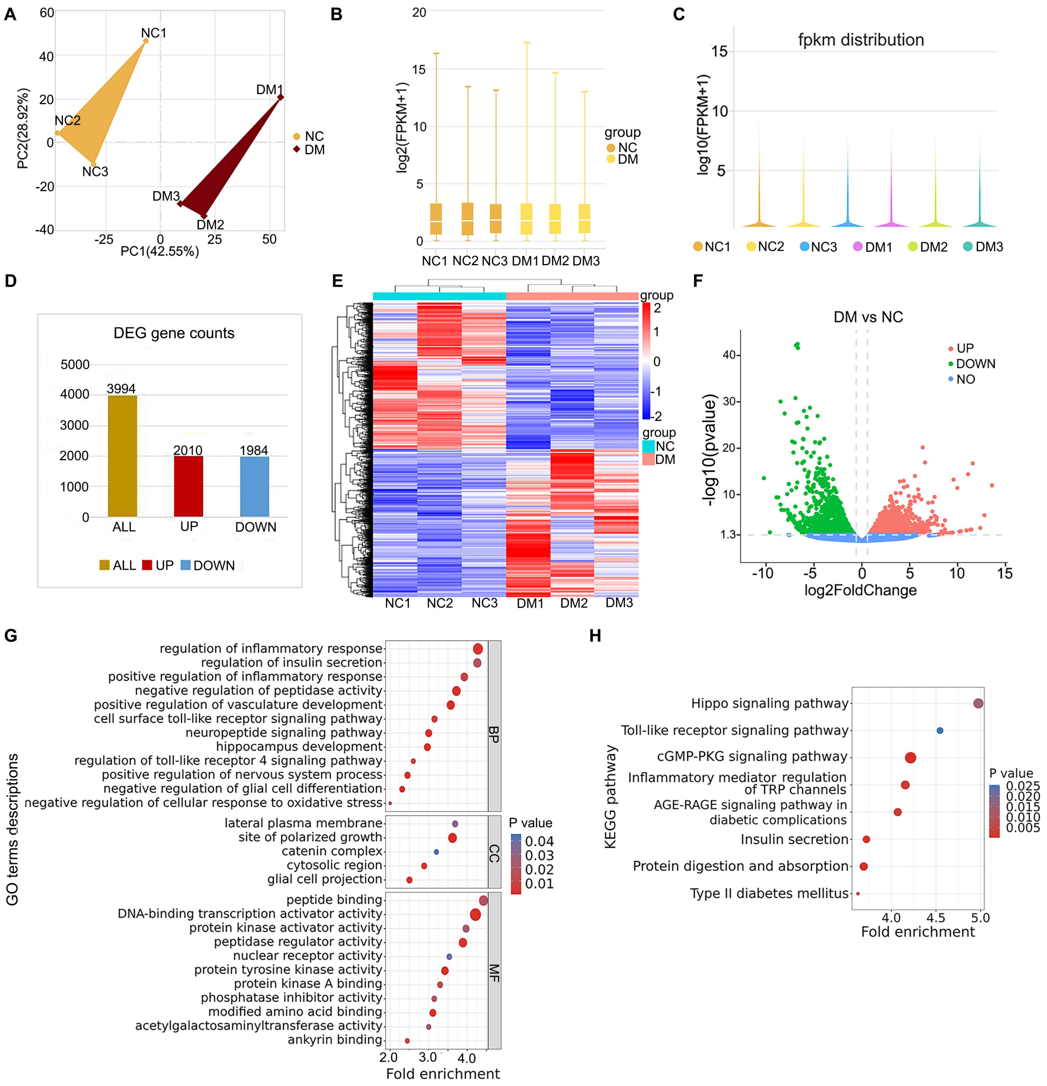


Fig. S6 Transcriptomic results in microglias cultured in different medium. (A) A plot illustrating the RNA quantitative PCA results for all samples. (B) The boxplot of gene expression distribution. (C) The violin plot of FPKM distribution. (D) Histogram of DEGs between two groups. (E) Heatmaps of selected DEGs in two groups, where light red represents high expression and blue represents low expression. (F) Volcano plots showing relative abundances of genes in two groups (G) GO enrichment in Transcriptome. (H) KEGG pathway analysis in Transcriptome. DM, diabetes mellitus; NC, negative control.

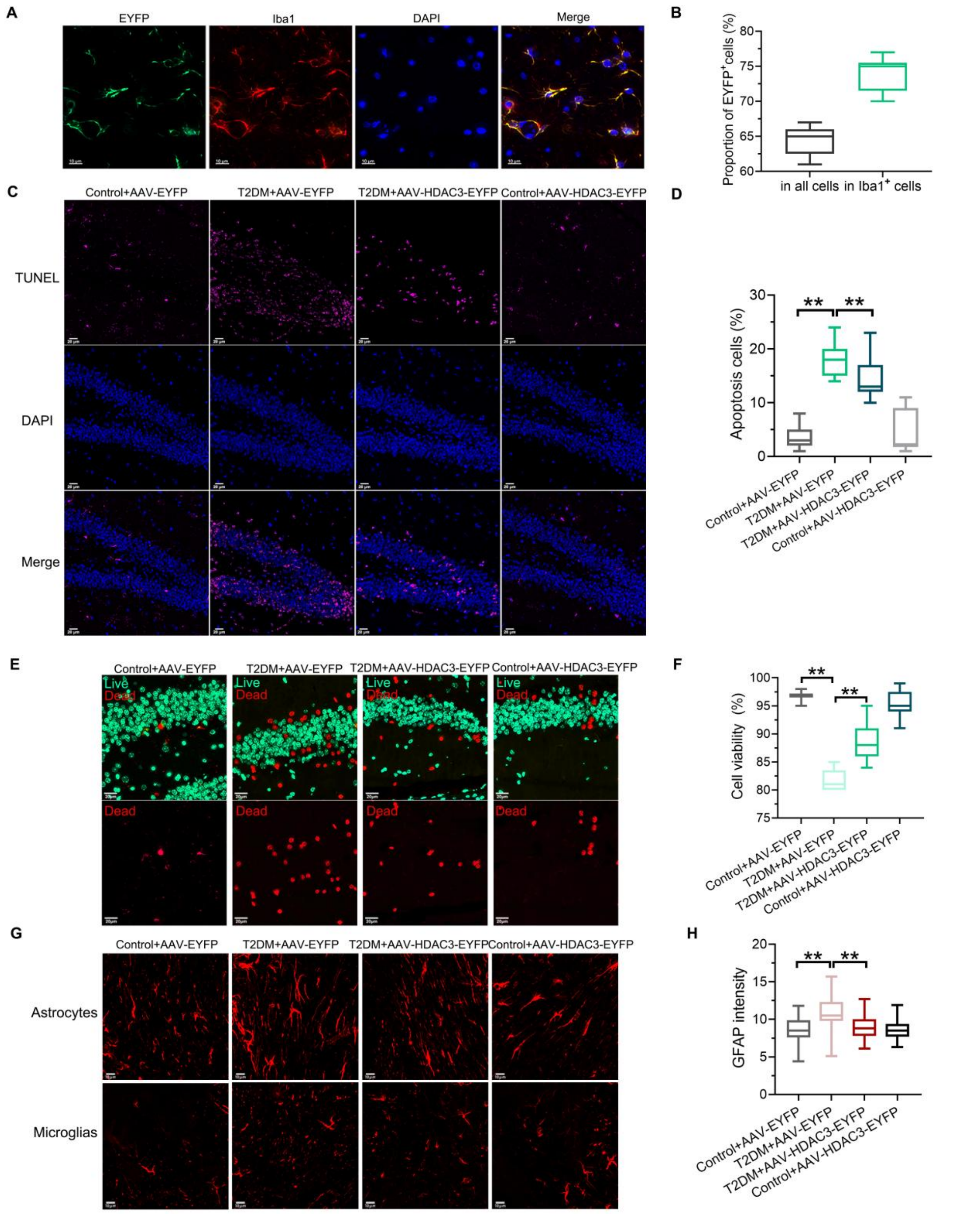


Fig. S7 Impact of AAV transduction on neuronal survival and gliosis in the hippocampus. (A) Representative immunofluorescence images showing the colocalization of EYFP and Iba-positive immunosignals in microglias of hippocampus transduced with AAV. Scale bar, 10 μm. (B) Quantitative analysis of AAV infection efficiency (n=3). (C) TUNEL assay detecting apoptotic cells. Scale bars: 20 μm. (D) Statistical quantification of TUNEL+ cells. (E) Viability assessment via Live/Dead

staining: viable (green) and non-viable (red) zones. Scale bars: 20 μm. (F) Cell viability of cells in hippocampus. (G) Fluorescence images of hippocampus sections expressing GFAP in microglias or astrocytes. Scale bar, 10 μm. (H) GFAP intensity of microglias or astrocytes in different groups. n=3 per group. Data are expressed as mean ± SD. Statistical analysis was performed using two-tailed Student's t tests or two-way ANOVA with Tukey test for differences among groups (B, D, F, and H). $^{**}P<0.01$.

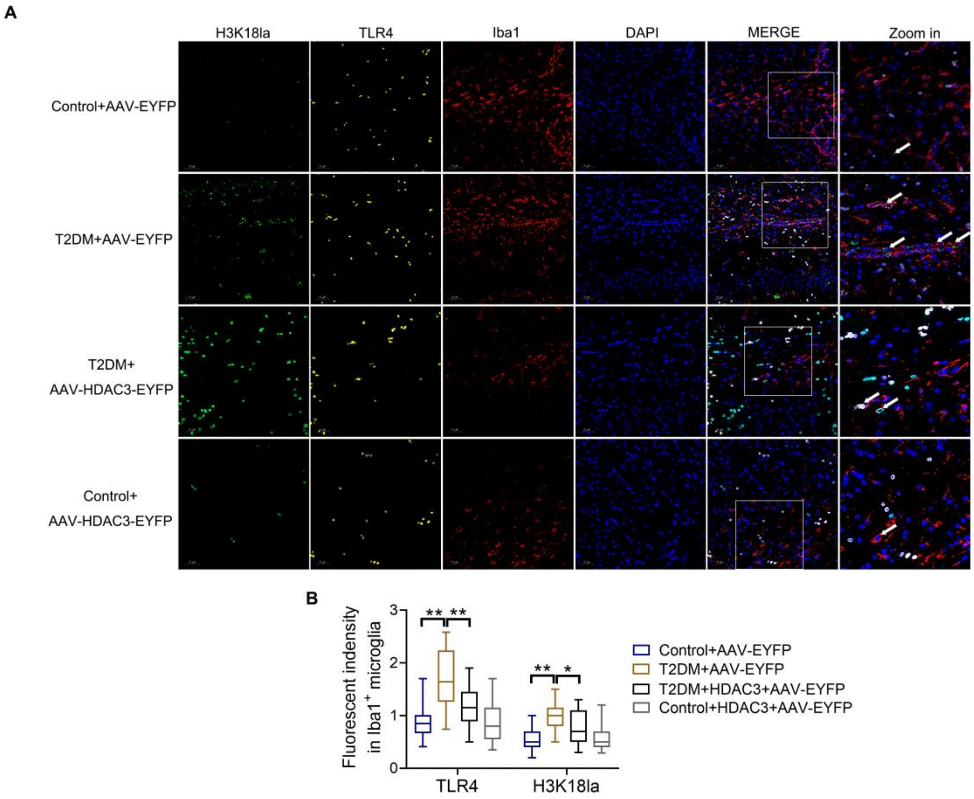


Fig. S8 Inhibition of lactylation of Histone and H3K18 leads to TLR4 suppression in immunofluorescence. (A) The representative images of immunofluorescence foci of H3K18la (green), TLR4 (yellow) and Iba1 (red) in hippocampal tissues from four groups of mice. (B) Quantitation of immunofluorescence for H3K18la and TLR4 in the Iba1+ microglia. n=3 per group. Data are expressed as mean ± SD. Statistical analysis was performed using two-tailed ANOVA with Tukey test for differences among groups. $^{**}P<0.01$, $^{*}P<0.05$.

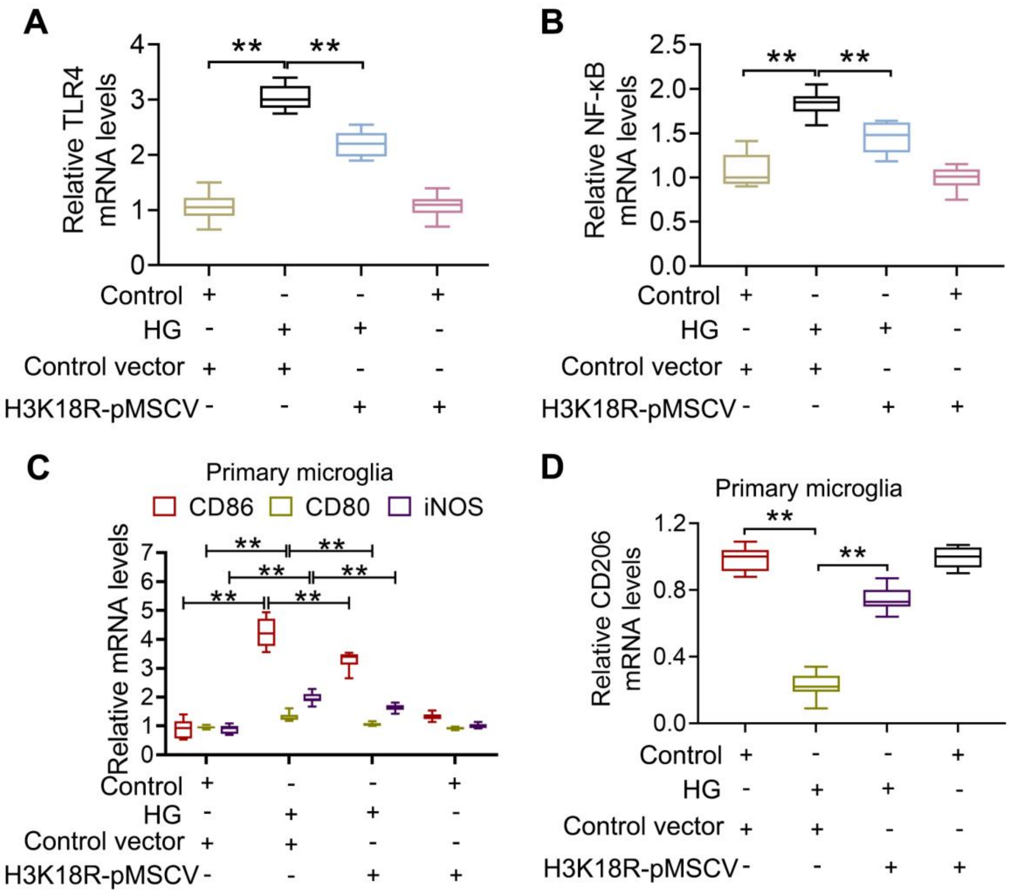


Fig.S9 Suppression of H3K18la inhibited TLR4 pathway and microglial M1 polarization. (A) mRNA expression for TLR4 in primary microglias. (B) mRNA expression for NF-κB in primary microglias. (C) mRNA expression for CD86, CD80 and iNOS in primary microglias. (D) mRNA expression for CD206 in primary microglias.n=3 per group. Data are expressed as mean ± SD. Statistical analysis was performed using two-way ANOVA with Tukey test for differences among groups. $^{*}P<0.05$, $^{**}P<0.01$.

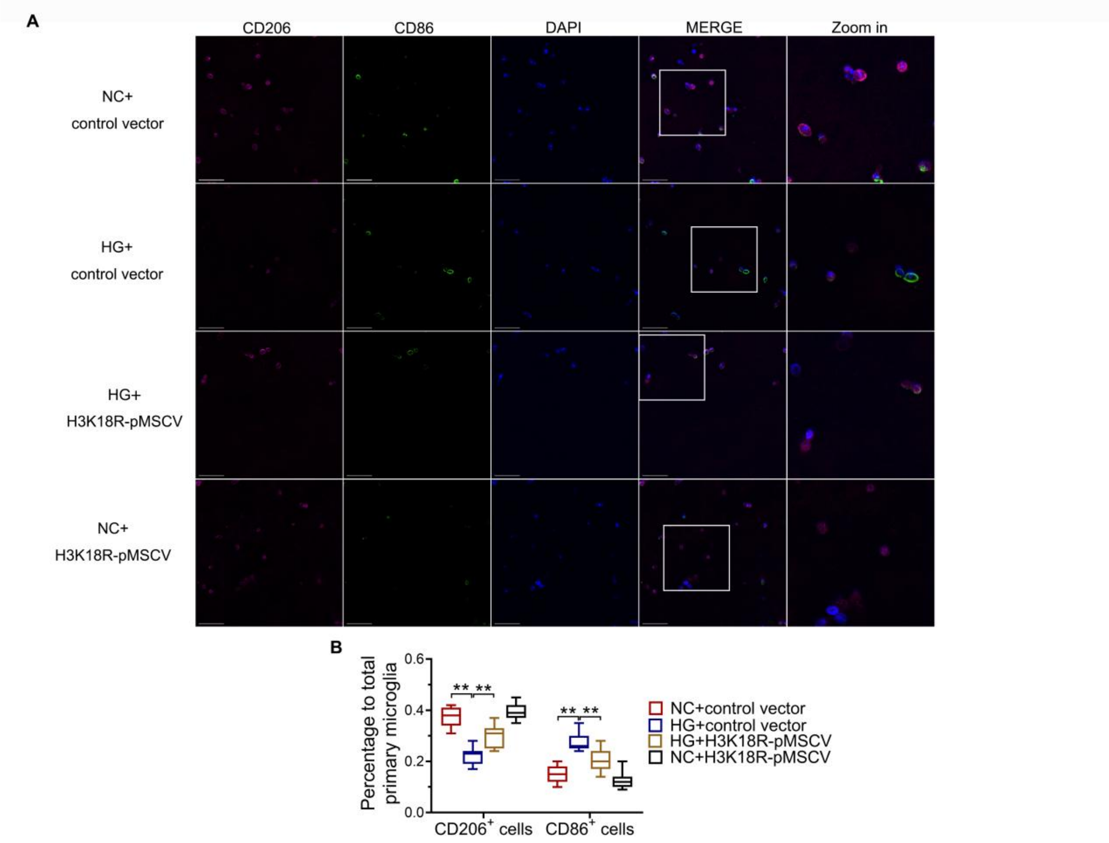


Fig. S10 Inhibition of lactylation of Histone and H3K18 leads to M2 microglia polarization in immunofluorescence. (A) Representative immunofluorescence images of CD86 (green), CD206 (pink) and nuclei (blue) after treated with H3K18R-pMSCV or Control vector in primary microglias cells. (B) Quantitation of immunofluorescence for CD206 and CD86 in primary microglias. n=3 per group. Data are expressed as mean ± SD. Statistical analysis was performed using two-tailed ANOVA with Tukey test for differences among groups. $^{**}P<0.01$, $^{*}P<0.05$.

# Supplementary Material-Uncropped blots

## Figure 2D

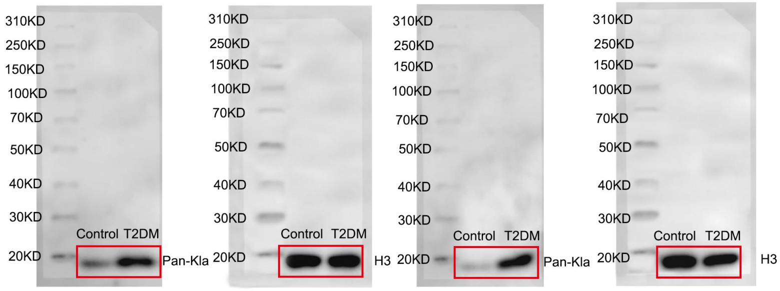


## Figure 5B

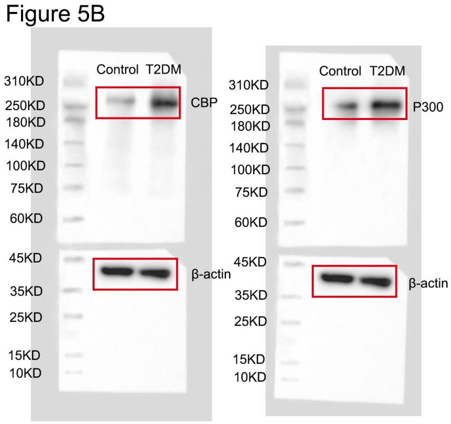


## Figure 5C

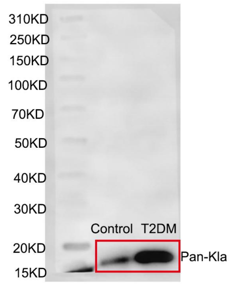


## Figure 5C

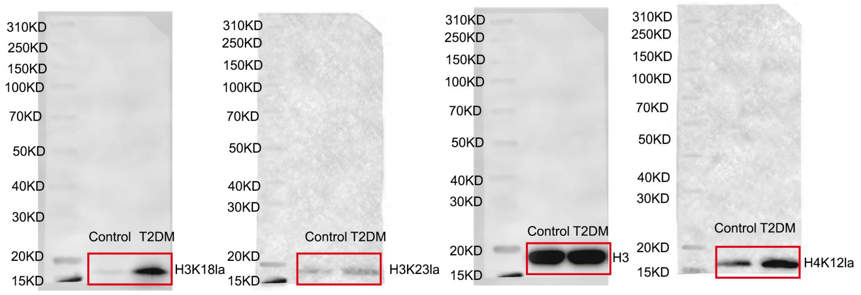


## Figure 7B

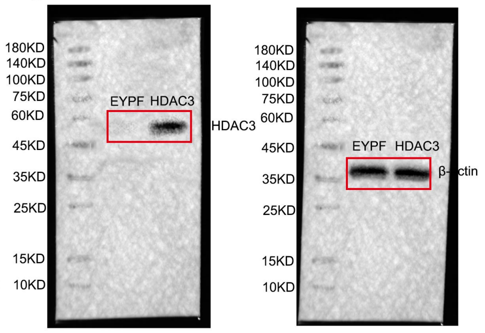


## Figure 7K

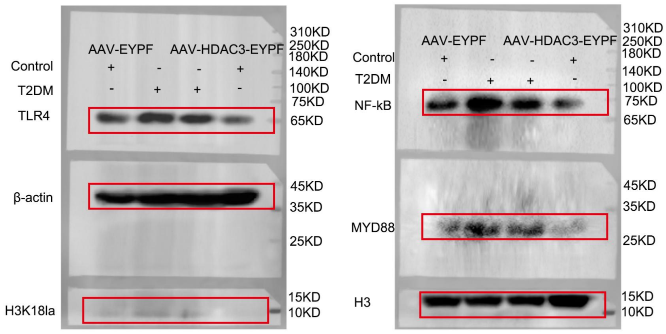


## Figure 8A

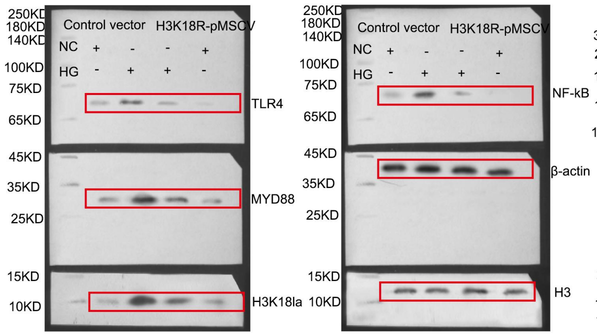


## Figure 8B

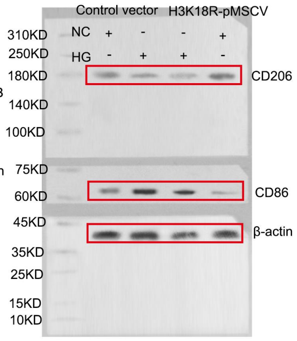


## Figure S2A

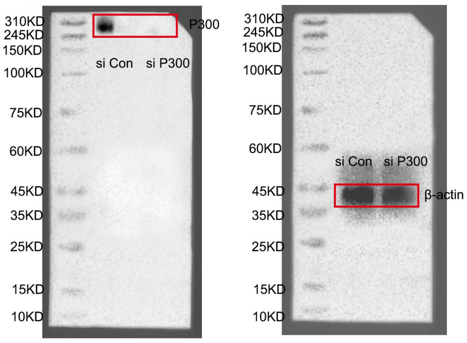


## Figure S2B

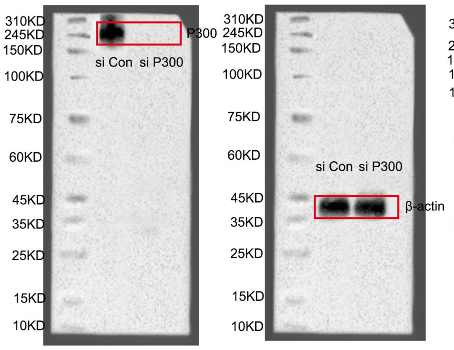


## Figure S3D

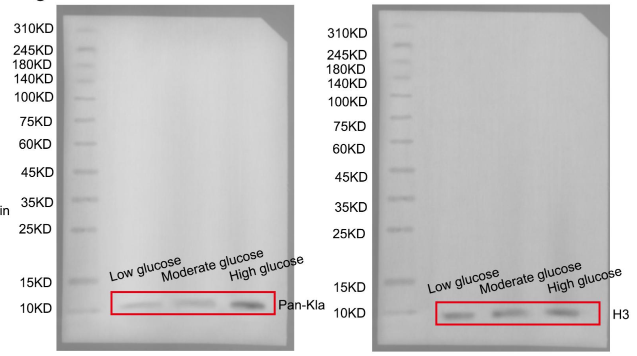

## Figure S3D continued

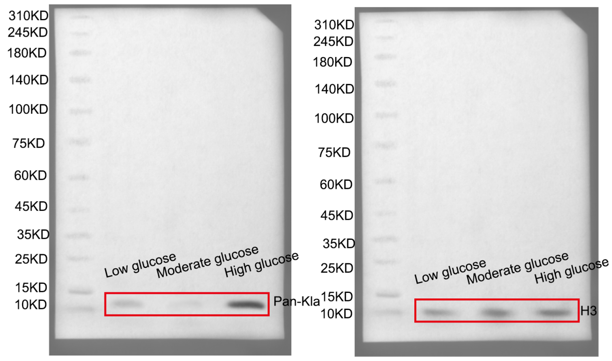


## Figure S4B

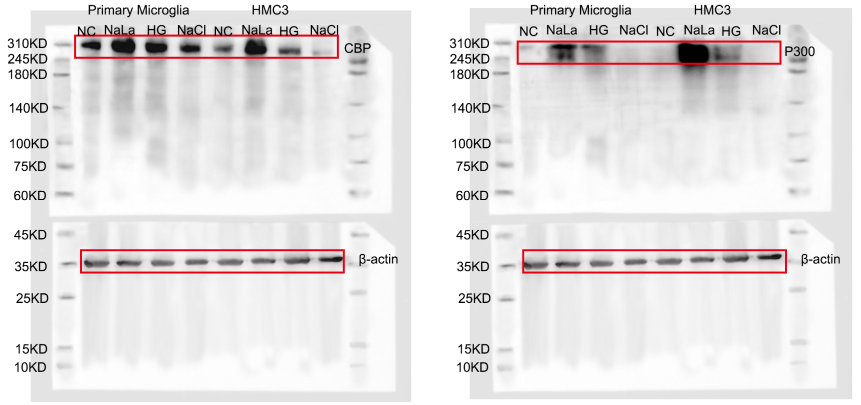


## Figure S4D

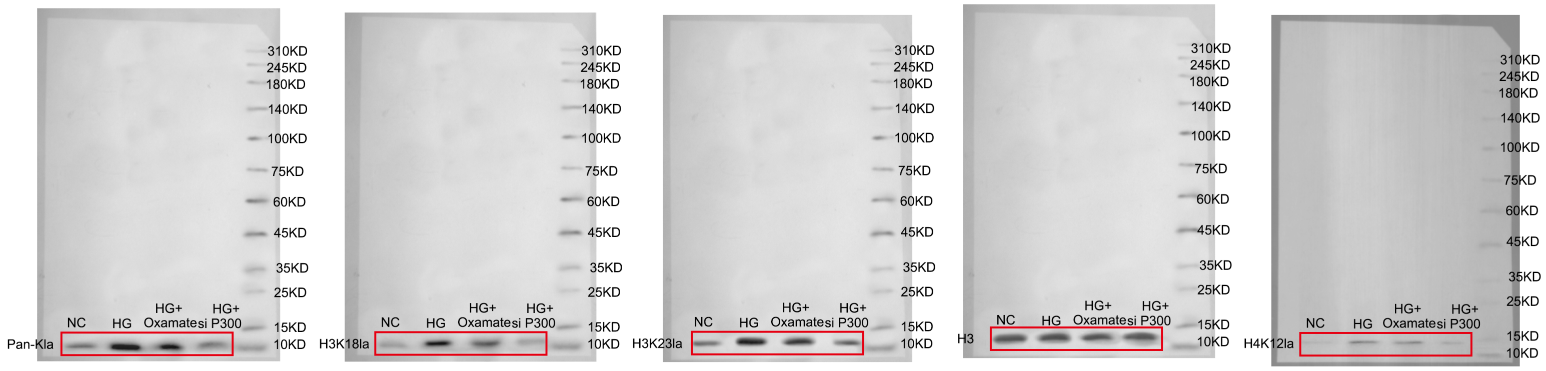


## Figure S4D continued

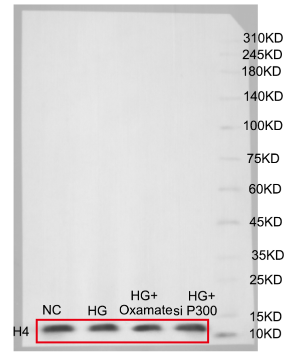


## Figure S4E

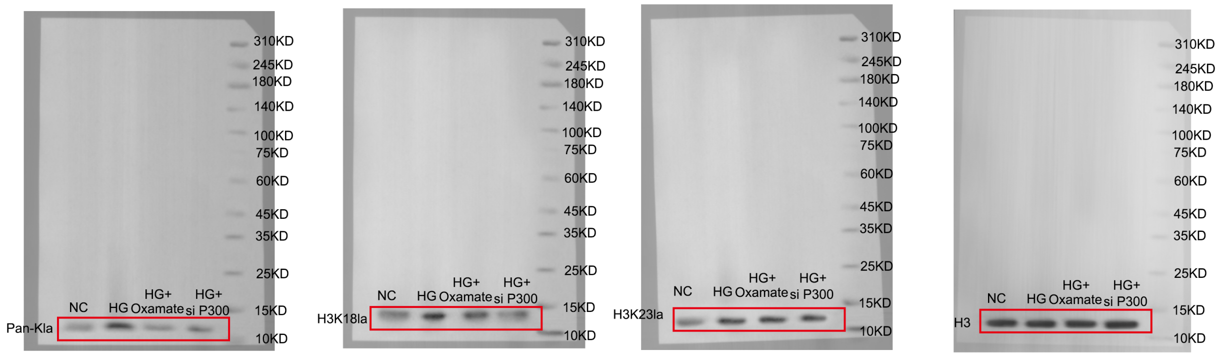


## Figure S4E continued

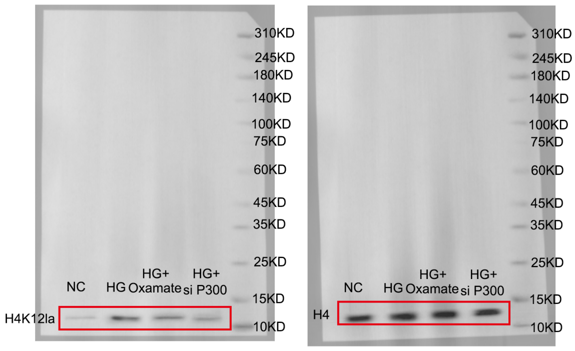


## Figure S5A

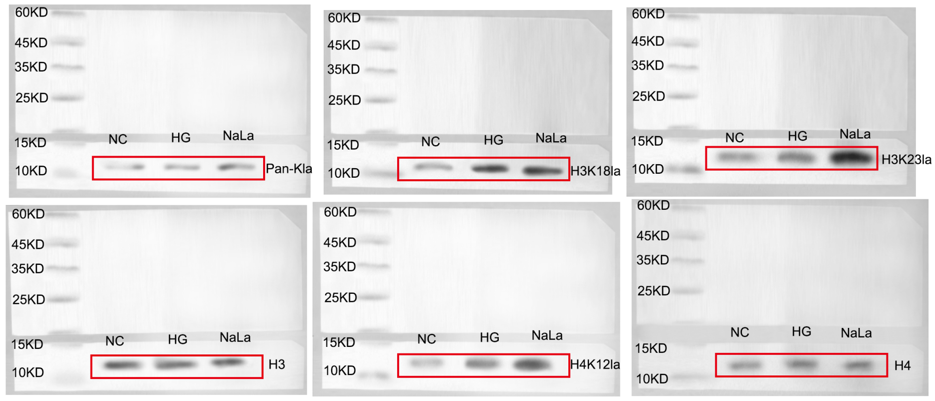

Figure S5B

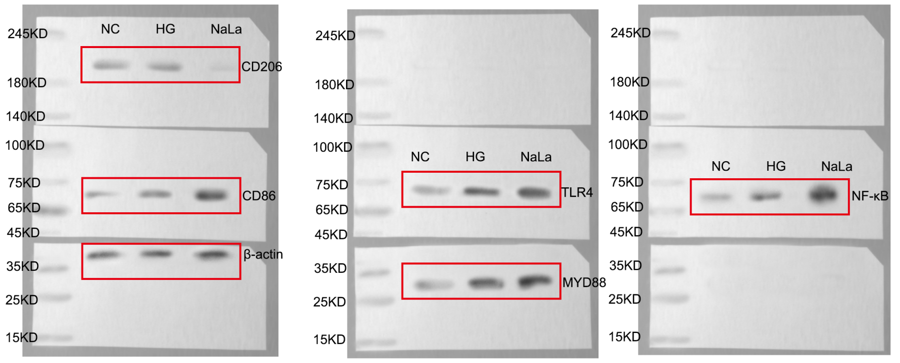


Figure S5C

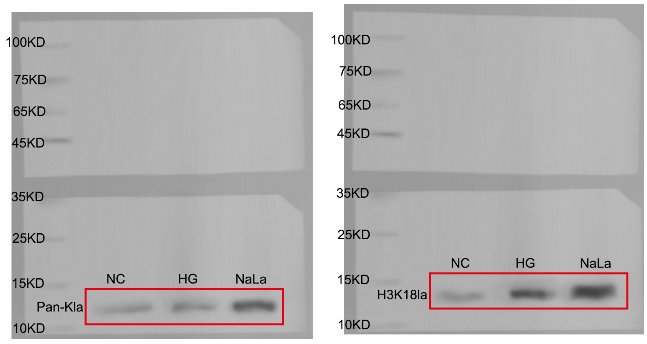


Figure S5C continued

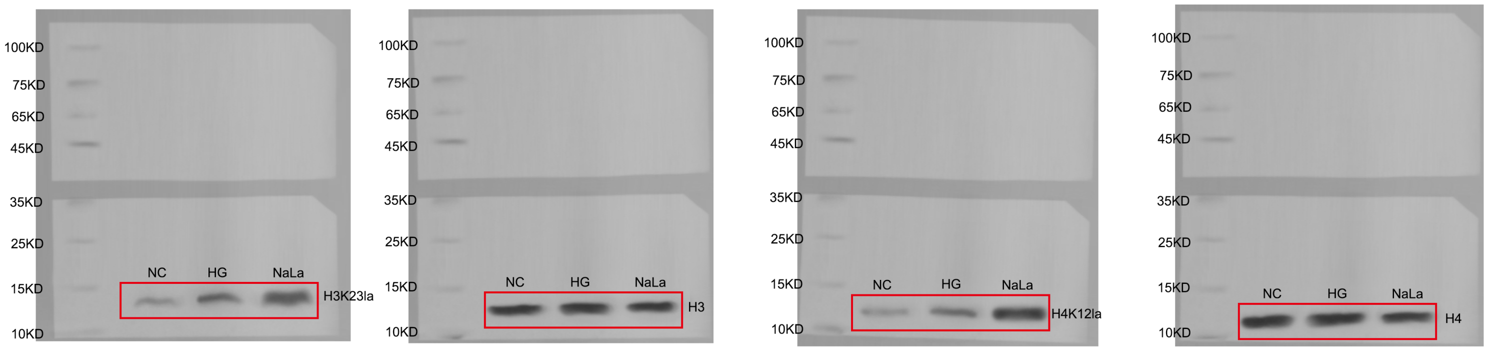


Figure S5D

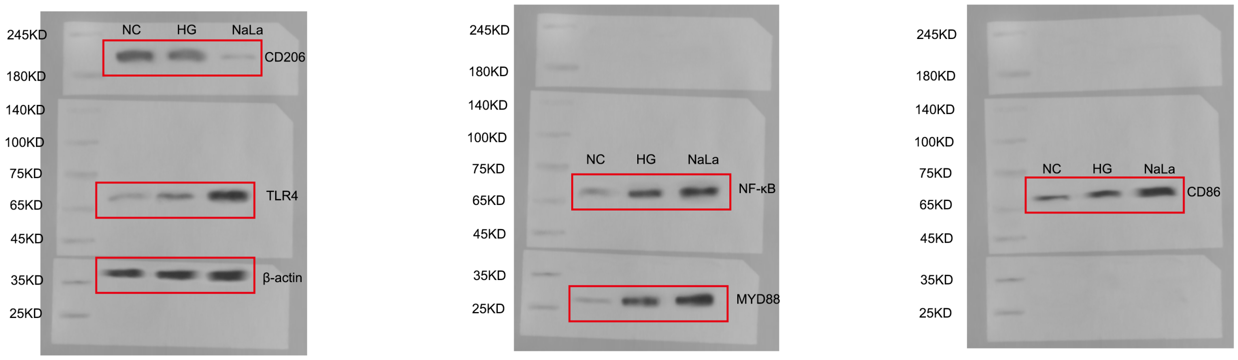

Table S1 Sequences of the primers for PCR.

| Gene name | For primary microglia | |
|---|---|---|
| | Forward (5'-3') | Reverse (5'-3') |
| TLR4 | TCGCTCAATCTGTCTTTCAC | TCTGACGATGTGGGTAGCG |
| NF-κB | CACTGCTCAGGTCCACTGTC | CTGTCACTATCCCGGAGTTCA |
| CD80 | TCAGTTGATGCAGGATACACCA | AAAGACGAATCAGCAGCACAA |
| CD86 | TCAATGGGACTGCATATCTGCC | GCCAAAATACTACCAGCTCACT |
| CD206 | GAGGGAAGCGAGAGATTATGGA | GCCTGATGCCAGGTTAAAGCA |
| CD68 | TCCACCCTCGCCTAGTCCAA | GCCCCAAGCCCTCTTTAAGC |
| iNOS | CCCTTCCGAAGTTTCTGGCAGCAGC | GGCTGTCAGAGCCTCGTGGCTTTGG |
| IL-1β | CTCACAAGCAGAGCACAAGC | CAGTCCAGCCCATACTTTAGG |
| TMEM119 | CCTTCACCCAGAGCTGGTTC | GGCTACATCCTCCAGGAAGG |
| P2RY12 | CATTGACCGCTACCTGAAGACC | GCCTCCTGTTGGTGAGAATCATG |
| β-actin | ACCTTCTACAATGAGCTGCG | CTGGATGGCTACGTACATGG |
| **Gene name** | **For HMC3 cells** | |
| | Forward (5'-3') | Reverse (5'-3') |
| TLR4 | CAAGAACATAGATCTGAGCTTCAACCC | GCTGTCCAATAGGGAAGCTTTCTAGAG |
| NF-κB | GCGAGAGGAGCACAGATACCACCAA | GGCAGATCTTGAGCTCGGCAGTGTT |
| CD80 | TGGTGCTGGCTGGTCTTTC | CTGTGCCACTTCTTTCACTTCC |
| CD86 | ACATTCTCTTTGTGATGGCCTTC | TGCAGTCTCATTGAAATAAGCTTGA |
| CD206 | CCACAGCATTGAGGAGTTTG | ACAGCTCATCATTTGGCTCA |
| iNOS | ACAACAAATTCAGGTACGCTGTG | TCTGATCAATGTCATGAGCAAAGG |
| β-actin | CCTGGCACCCAGCACAAT | GGGCCGGACTCGTCATAC |

Table S2 The siRNA or vector sequences

| | For primary microglia | |
|---|---|---|
| | Forward (5’-3’) | Reverse (5’-3’) |
| si P300 | CCAGAUGAAUUAAUCAACUTT | AUUAUCUUGUGGCUGCUGCTT |
| H3K18R | CAAGGCGCCACGCAGACAGTT GGCCAC | GTGGCCAACTGTCTGCGTGGCG CCTTG |
| | **For HMC3 cells** | |
| | Forward (5’-3’) | Reverse (5’-3’) |
| si P300 | CAGAGCAGUCCUGGAUUAGTT | CUAAUCCAGGACUGCUCUGTT |

Table S3 The record of weight and serum biochemical analyses of mice

| Characteristics | Controls (n=30) | T2DM (n=30) |
|---|---|---|
| **Weight (g)** | 30.7±1.5 | 31.8±1.1.7[##] |
| **Fasting glucose (mmol/l)** | 5.6±1.0 | 16.2±2.3[#] |
| **Triglyceride (mg/ml)** | 1.3±0.3 | 1.5±0.4[#] |
| **Free Fatty Acids (mg/ml)** | 0.03±0.005 | 0.03±0.006[#] |

[#]P<0.05, [##]P<0.01 vs. Control group.
