# Supplementary material for: H3K18 lactylation potentiates microglial polarization via the TLR4 pathway in diabetes-induced cognitive impairment
Source: JCI Insight. 2025 Nov 4;10(24):e188077. doi: 10.1172/jci.insight.188077 (PMC12890480; doi:10.1172/jci.insight.188077)

Supplementary Material-Uncropped blots

Figure 2D

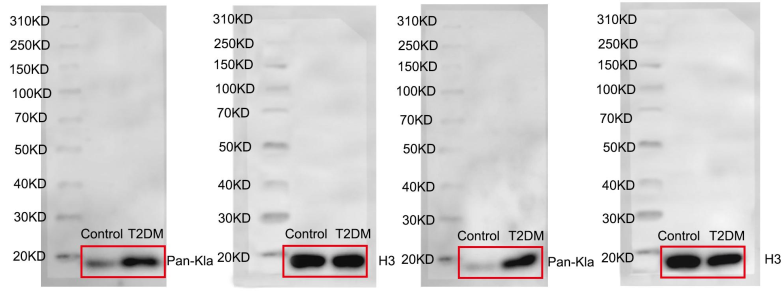

Figure 5B

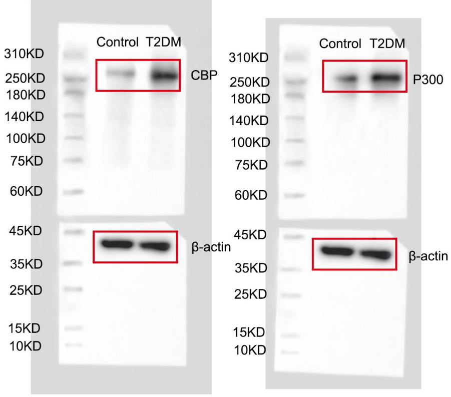

Figure 5C

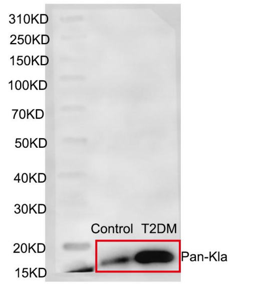

Figure 5C

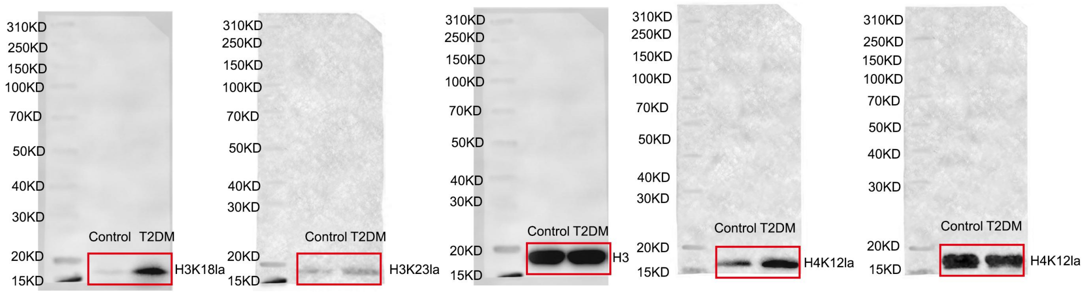

Figure 7B

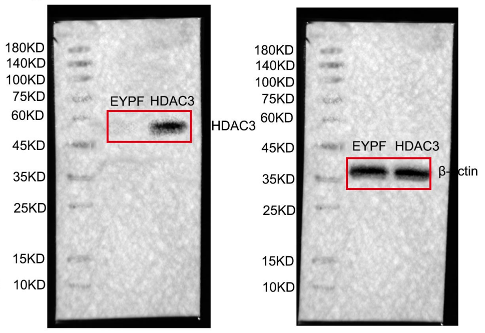

Figure 7K

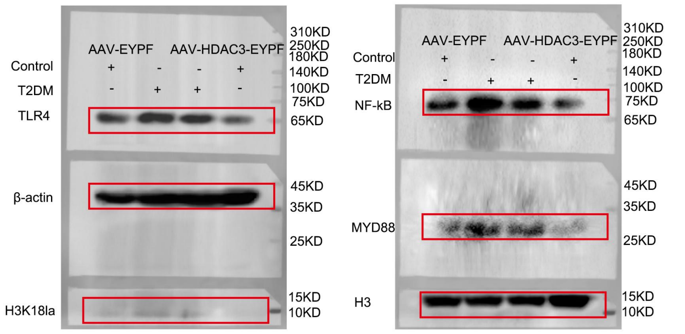

Figure 8A

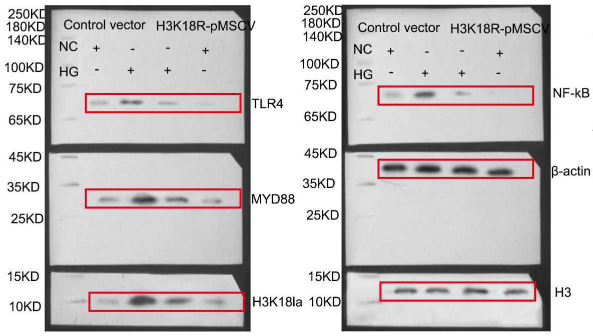

Figure 8B

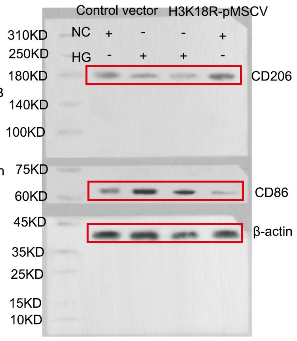

Figure S2A

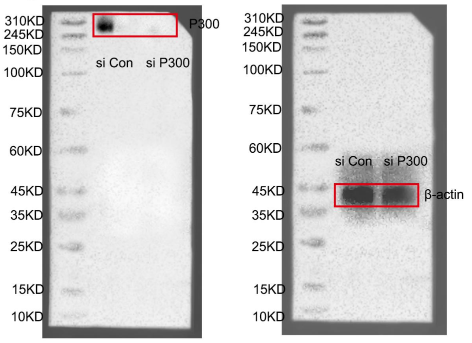

Figure S2B

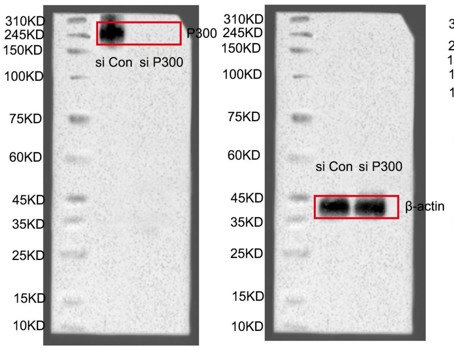

Figure S3D

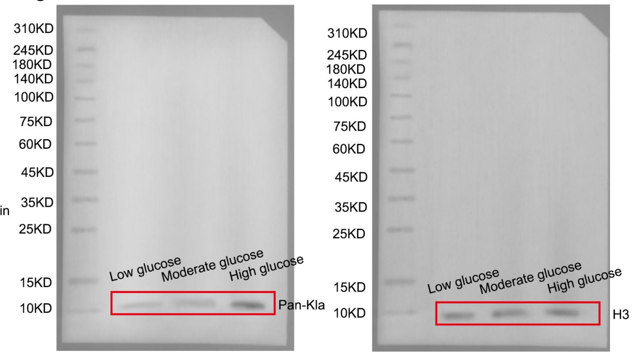

Figure S3D continued

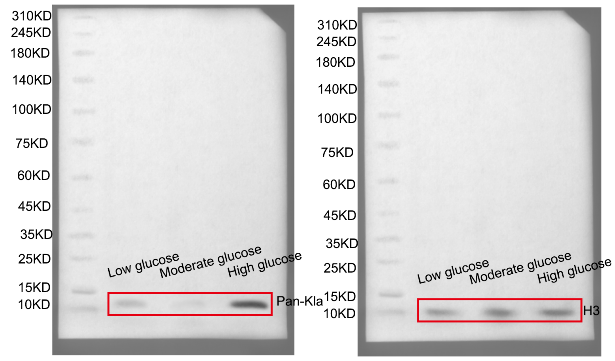

Figure S4B

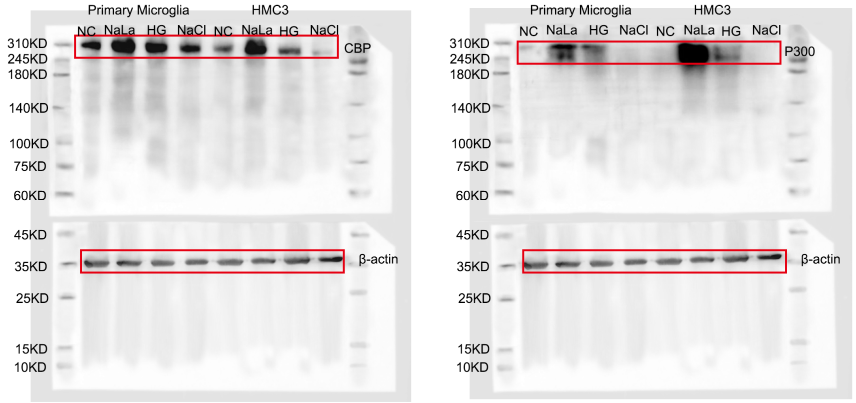

Figure S4D

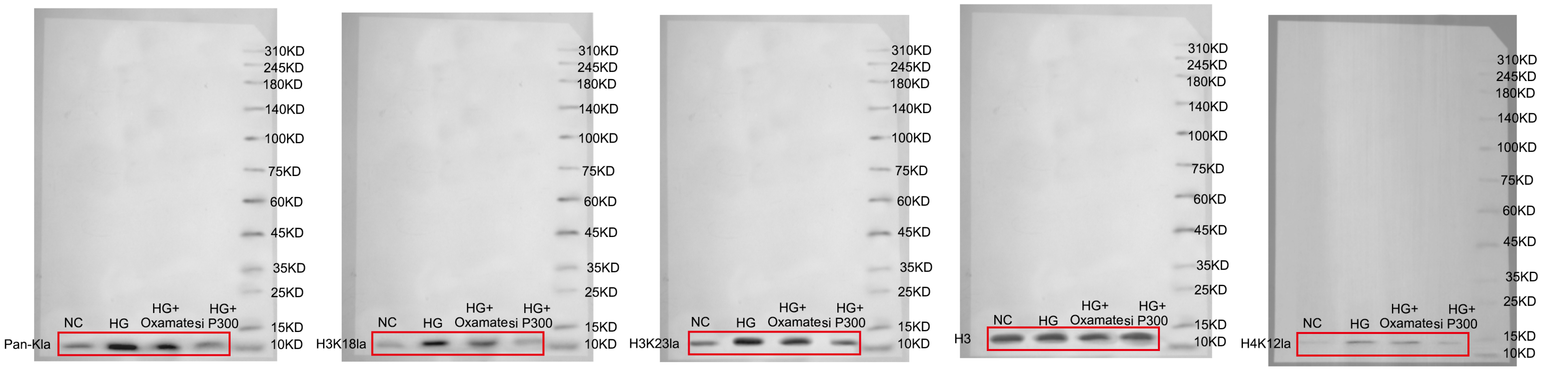

Figure S4D continued

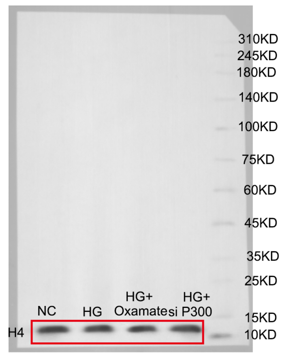

Figure S4E

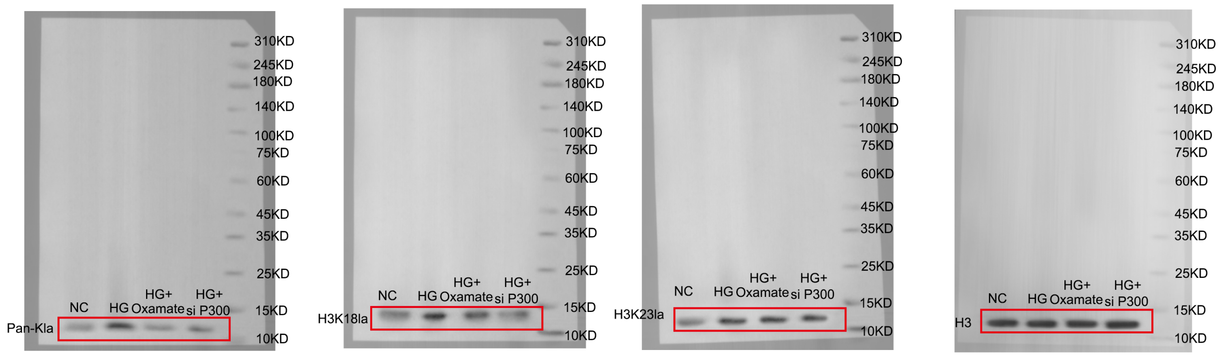

Figure S4E continued

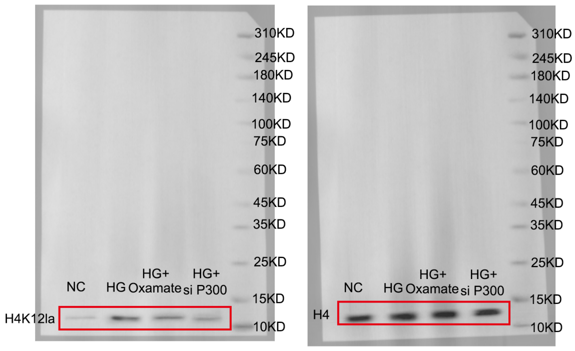

Figure S5A

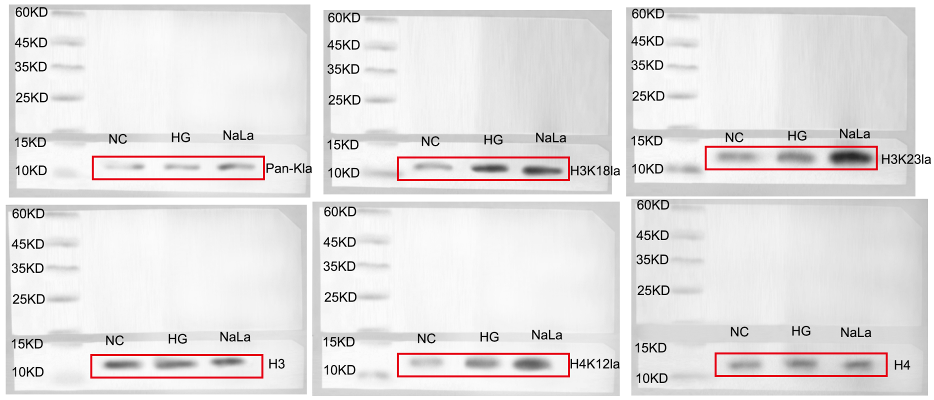

Figure S5B

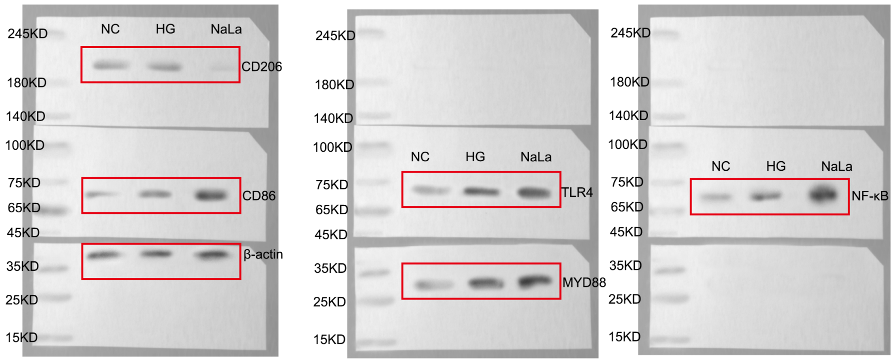

Figure S5C

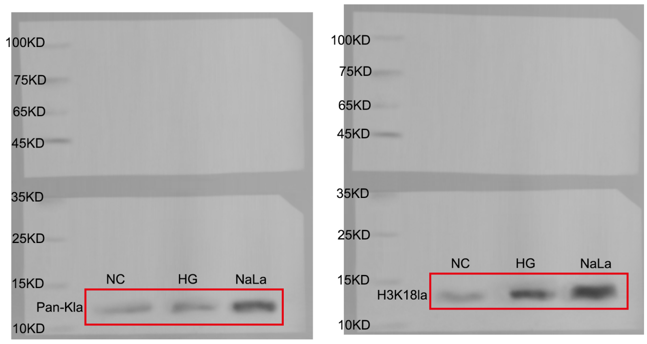

Figure S5C continued

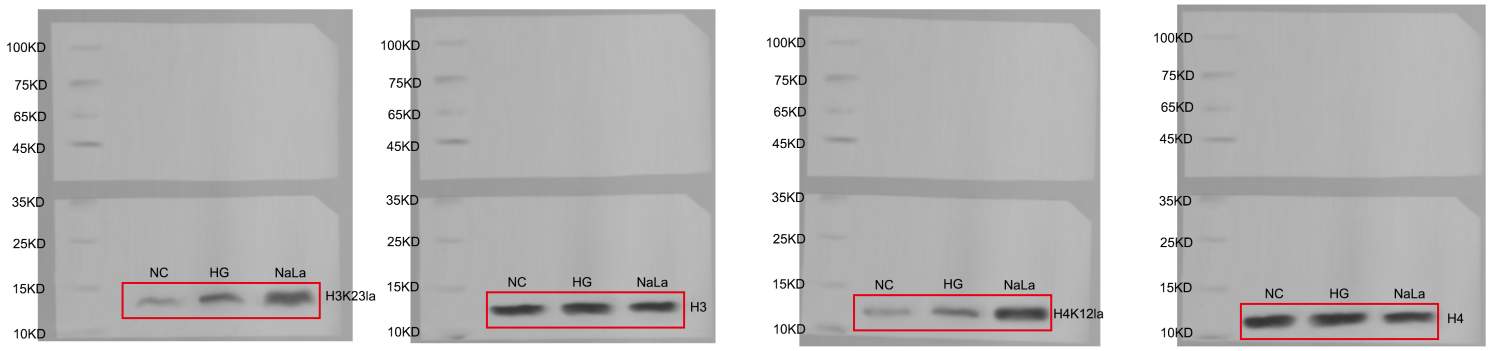

Figure S5D

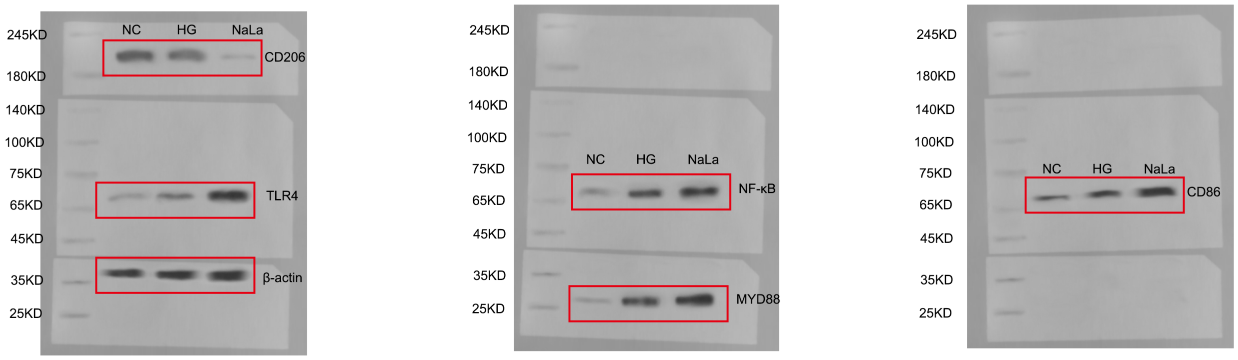

Supplement: Unedited blot and gel images [file jciinsight-10-188077-s142.pdf]
